# Polymeric Hydrogels for Controlled Drug Delivery to Treat Arthritis

**DOI:** 10.3390/pharmaceutics14030540

**Published:** 2022-02-28

**Authors:** Anuradha Gupta, Jungmi Lee, Torsha Ghosh, Van Quy Nguyen, Anup Dey, Been Yoon, Wooram Um, Jae Hyung Park

**Affiliations:** 1 School of Chemical Engineering, College of Engineering, Sungkyunkwan University, Suwon 16419, Korea; anuradhagupta01@gmail.com (A.G.); norani@hanmail.net (J.L.); ghosh21torsha@gmail.com (T.G.); vquynguyents@gmail.com (V.Q.N.); anupdey92@gmail.com (A.D.); yb9334@gmail.com (B.Y.); tings0609@nate.com (W.U.); 2 Biomedical Institute for Convergence at SKKU (BICS), Sungkyunkwan University, Suwon 16419, Korea

**Keywords:** arthritis, polymeric hydrogels, protein/polysaccharide polymers, synthetic polymers, hybrid hydrogel composite, nanoparticles/microparticles-embedded hydrogels

## Abstract

Rheumatoid arthritis (RA) and osteoarthritis (OA) are disabling musculoskeletal disorders that affect joints and cartilage and may lead to bone degeneration. Conventional delivery of anti-arthritic agents is limited due to short intra-articular half-life and toxicities. Innovations in polymer chemistry have led to advancements in hydrogel technology, offering a versatile drug delivery platform exhibiting tissue-like properties with tunable drug loading and high residence time properties This review discusses the advantages and drawbacks of polymeric materials along with their modifications as well as their applications for fabricating hydrogels loaded with therapeutic agents (small molecule drugs, immunotherapeutic agents, and cells). Emphasis is given to the biological potentialities of hydrogel hybrid systems/micro-and nanotechnology-integrated hydrogels as promising tools. Applications for facile tuning of therapeutic drug loading, maintaining long-term release, and consequently improving therapeutic outcome and patient compliance in arthritis are detailed. This review also suggests the advantages, challenges, and future perspectives of hydrogels loaded with anti-arthritic agents with high therapeutic potential that may alter the landscape of currently available arthritis treatment modalities.

## 1. Introduction

Joint diseases such as rheumatoid arthritis (RA) and osteoarthritis (OA) involve the inflammatory immune response at both local (joint site) and systemic levels, characterized by inflammatory damage to cartilage and bones, resulting in severe articular joint pain and reduced joint mobility [1,2]. To treat arthritis, hydrogel-mediated delivery of therapeutic agents is at the forefront of therapeutic invention, offering safe and efficacious therapies by precise and controlled drug delivery to target sites [3].

Therapeutic strategies employing non-steroidal anti-inflammatory drugs (NSAIDs), glucocorticoids (GCs), disease modifying anti-rheumatic drugs (DMARDs), immunotherapeutic agents (i.e., antisense oligonucleotides (ASOs), cytokine inhibitors such as TNF-α inhibitors and nuclear factor-κB inhibitors etc.), and cells have been investigated to relieve pain and control inflammation in arthritis. However, their long-term use and clinical application are limited because of high systemic toxicity and limited therapeutic efficacy [4]. Localized drug administration via intra-articular injection has been attempted to improve drug bioavailability at the joint site and reduce systemic toxicity [5,6] However, due to the rapid efflux of drugs via systemic circulation following injection, maintaining the therapeutic drug concentration for prolonged periods has been challenging. Novel drug delivery strategies employing polymer–drug conjugates, polymeric nanocarriers (i.e., nanoparticles (NPs) or liposomes), and protein and peptide-based delivery systems have overcome most of the issues related to conventional dosage forms [7,8]. However, particulate delivery systems have shown difficulties in localized delivery at the target site, presenting low drug loading and drug release efficiency. Liposomes posed difficulties in achieving sustained drug release with hydrophobic drugs and maintaining formulation stability. On the other hand, nanoparticles (NPs) and microparticles (MPs) displayed rapid clearance from the joint site via sub-synovial capillaries and lymphatics and via macrophages, respectively. Moreover, particulate delivery systems also suffer from limitations in cell delivery, maintenance of their phenotype at the joint site, and their inability to mimic the extracellular matrix (ECM) microenvironment. In the same context, engineered hydrogel composites represent a major unmet need for drug and cell delivery to bone and cartilage tissue, and have emerged as a depot to form versatile drug delivery platforms by overcoming most of the barriers posed by former approaches to treat inflammatory arthritis. Hydrogels are 3-dimensional (3D) networks constructed by the cross-linking of hydrophilic polymer chains with high water content, allowing for the efficient exchange of oxygen and nutrients, providing structural support for cell growth and tissue-like properties. Hydrogels have demonstrated several advantages in tissue engineering applications, which include:

- Capacity to deliver multiple agents such as drugs, oligonucleotides, monoclonal antibodies, growth factors, and cells (chondrocytes and mesenchymal stem cells; MSCs);
- Ability of in situ-forming hydrogels to maintain a concentrated form of drug (depot) for a long period of time following intra-articular injection, thus eliminating the need for repetitive dosing (injections);
- Capacity to provide minimally invasive or non-invasive treatment options with injectable/transdermal drug delivery to treat arthritis, displaying high therapeutic efficacy and manageable toxicity at a low dose, thereby improving patient compliance;
- The ability of the hydrogel structure to mimic the extracellular matrix (ECM) microenvironment, permitting efficient tissue regeneration. Following application, hydrogels maintain hydrated structures, and are therefore suited for cell delivery applications. Moreover, hydrogels can be modified with ligands to enhance their interaction with chondrocytes and/or their progenitor cells for cartilage tissue engineering (CTE) [9].

**Figure 1:**
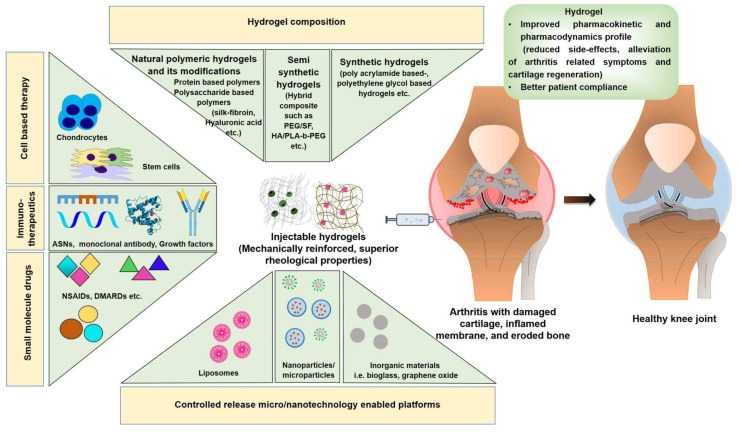
Overview of polymeric hydrogels used to deliver small molecule drugs, immunotherapeutics, and cells to joint sites for alleviating pain, swelling, inflammation, thus providing viscoelastic, lubrication, and cartilage repair advantages.

Several natural and/or synthetic polymers have been investigated either separately or in their cross-linked form to formulate hydrogels. Cross-linking allows for the development of novel engineered hydrogel materials with appropriate release characteristics and high physical integrity. This review highlights the advantages and drawbacks of polymeric materials (natural, synthetic, and semi-synthetic). Figure 1 shows the different types of polymeric materials used to prepare hydrogel and hydrogel-encapsulated therapeutic agents with the aim of improving the pharmacokinetic and pharmacodynamic responses in arthritis. Representative examples from each category of polymeric materials used to prepare hydrogels are also discussed from the perspective of material sections, along with their features, drawbacks, and recent advancements. Furthermore, the incorporation of micro-and nanotechnologies in hydrogels for efficient and long-term delivery of therapeutic agents (small molecules, immunotherapy, and cell-based therapies) is also discussed.

## 2. Hydrogel Composition: Materials Used to Prepare Hydrogels

Hydrogels are prepared using the sol–gel transition phenomenon due to which they possess unique physical properties such as high sorption capacity, porous structure, biocompatibility, bioadhesion, sufficient mechanical strength, and tissue-like mechanical properties. These properties make them suitable for delivering drugs and cells, resulting in efficient cartilage and bone tissue regeneration, and/or symptomatic relief from arthritis. Furthermore, controlling the release of therapeutics can be facilitated through polymer modifications such as charge modification and by altering the nature and density of cross-linking. Hydrogels can be cross-linked via physical or chemical cross-linking methods. Physical cross-linked hydrogels exhibit reversible sol–gel transitions and are often sensitive to changes in temperature or ion concentration. Chemical cross-linking methods involve specific cross-linking reactions such as Michael addition [10], Schiff-base reaction [11], and click chemistry (azide-alkyne cycloaddition) [12].

An ideal hydrogel should exhibit a short gelation time, high water content, controlled and slow degradation, pH stability, and sufficient mechanical properties to withstand the compressive and shear stresses of the joint, where natural and synthetic hydrogel scaffolds have displayed mechanical strength in the range of 0.45–5.65 MPa and 15–125 MPa, respectively [13]. Therefore, different polymeric materials, either separately or in combination, are used to meet these criteria. In general, polymeric materials can be categorized as natural, synthetic, or semi-synthetic. Naturally derived polymers are mainly peptide/protein-based, polysaccharide-based, or a combination of proteins and polysaccharides.

Synthetic polymers are highly plastic, produced by the chemical synthesis of repetitive monomer units or obtained by cross-linking different monomer units.

### 2.1. Hydrogels Prepared Using Naturally Derived Material

**Figure 2:**
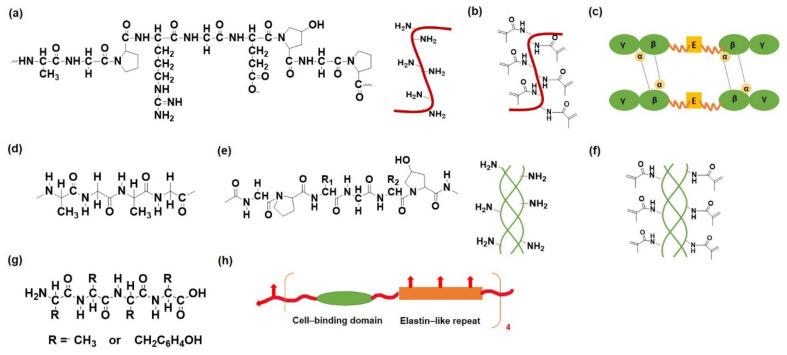
Chemical structure of protein/peptide–based polymers and their modifications (**a**) gelatin, (**b**) methacrylated gelatin (GelMA), (**c**) fibrin (protein structure), (**d**) silk fibroin (primary chemical structure and protein structure, (**e**) collagen, (**f**) methacrylated collagen, (**g**) silk-sericin, and (**h**) elastin-like protein.

Naturally derived polymers are biocompatible and biodegradable and provide adhesive surfaces for cell attachment. These are hydrophilic in nature, present excellent swelling properties, and may regulate various signaling pathways. The by-products generated by the degradation of naturally derived polymers are also nontoxic. However, hydrogels prepared from natural polymers suffer from poor mechanical strength and accelerated degradation characteristics. Furthermore, it is difficult to manage batch-to-batch variation in order to control the molecular weight range of natural polymers [14]. Natural polymeric materials can be classified as peptide/protein-based, polysaccharide-based, and protein–polysaccharide-based polymers. Herein, we discuss natural polymeric materials, features, and applications, and provide insight into different polymeric modifications and cross-linking. Figure 2 shows the chemical structure of the protein/peptide-based polymers and their modifications for preparing the hydrogels.

#### 2.1.1. Protein and Peptide-Based Hydrogels

##### Gelatin

Gelatin is a high molecular weight polypeptide derived from the partial hydrolytic degradation of collagen, and contains a large number of tripeptide (arginine–glycine–aspartic acid; RGD) sequences. The tripeptide facilitates integrin binding sites and thus favors cell adhesion and proliferation. In addition to being easily obtainable from natural sources, gelatin has shown additional advantages such as favorable structural properties, high water uptake ability, thermo-reversibility, biocompatibility, biodegradability, cost-effectiveness, and low antigenicity. Therefore, gelatin-based biomaterials have been frequently used as drug delivery systems and hydrogel scaffolds for artificial skin, bone grafts, wound dressings, and CTE applications [15,16]. Gelatin-based hydrogels can be easily prepared by physical, chemical, and enzymatic cross-linking methods. Despite their advantages, gelatin-based hydrogels have certain shortcomings such as poor mechanical strength, low delivery efficacy, and difficulty in controlling drug release. To compensate for these shortcomings, modified gelatin hydrogels such as methacrylated gelatin (GelMA) [17]) and hybrid gelatin hydrogels prepared by cross-linking gelatin with natural polymers such as gelatin–hyaluronic acid [18], gelatin–chitosan [19], and gelatin–alginate [20] or with synthetic polymers such as gelatin–polyacrylamide [21] and gelatin–poly(vinyl alcohol) (PVA) [22] have been developed.

GelMA hydrogels can be easily prepared via in situ photo-cross-linking methods and demonstrate superior mechanical properties over gelatin hydrogels. Hybrid GelMA-HA hydrogels prepared via the photo-cross-linking of GelMA and methacrylated HA (HAMA) displayed 2-fold higher mechanical properties than GelMA, augmented chondrogenesis of human bone marrow-derived MSCs (hBMSCs), and promoted both cartilage and subchondral bone regeneration in animal models of OA [18]. Furthermore, in order to omit the need for a photoinitiator that may sometimes pose toxicity and to modulate the mechanical properties, gelatin–PVA hydrogels were fabricated via a chemical cross-linking/freeze–thaw method, and they displayed 4-fold higher compressive strength and 5-fold higher Young’s modulus compared to that of gelatin [22].

##### Fibrin

Fibrin is a fibrous plasma protein that aids in blood coagulation. The polymerization/gelation of fibrin is achieved by the action of the proteolytic enzyme thrombin on fibrinogen to produce the monomer fibrin, which exhibits mechanical properties similar to those of blood clots. As a natural protein, fibrin has demonstrated excellent viscoelastic properties, biocompatibility, and biodegradability. Fibrin-based hydrogels are easily degraded by the action of the fibrinolytic system. Fibrin can bind to cells, ECM proteins, and growth factors. Because of its adhesive property, fibrin is mostly used as a sealant and scaffold for wound treatment and for the maintenance of hemostasis. By controlling the ratio of fibrinogen and thrombin, presence or absence of plasma proteins, ionic concentration, and reaction temperature, the porosity and mechanical strength of the fibrin hydrogel can be easily tuned. Notably, fibrin gel has presented low mechanical stiffness, faster degradation, and narrow stability (denaturation at high temperature and with the use of organic solvents), limiting its use as a single component hydrogel [23,24]. In order to overcome these drawbacks, fibrin is mostly used in combination with HA to prepare fibrin–HA hydrogel, delivering gapmers, cells, and even drug-loaded nanocapsules [25,26,27].

##### Collagen

Collagen, the raw material of gelatin and the main protein component of ECM, has shown advantages of biocompatibility, biodegradability, and low antigenicity. However, collagen hydrogels are very soft, easily contracted, and can rapidly degrade. Huang et al. developed a collagen hydrogel loaded with MSCs and demonstrated chondrogenesis with increased *Sox9* expression in arthritic animals [28]. To further potentiate the curative effects of collagen, a three-phase hydrogel composed of collagen, chondroitin sulfate, and HA was developed, and chondrocytes were seeded in the hydrogel. Both in vitro and in vivo studies have reported enhanced chondrogenesis with increased expression of cartilage-specific regulators such as *type II collagen*, *aggrecan*, and *Sox9*. These results postulate that the developed three-phase hydrogel can be used as an alternative to the collagen “gold standard”, showing higher stiffness properties than collagen. The three-phase hydrogel may be developed as a promising functional bioactive material for tissue regeneration [29].

##### Silk Fibroin

Along with the aforementioned advantages of natural polymers, silk fibroin (SF) has attracted attention because of its excellent mechanical properties (raw silk fiber has a tensile strength of 300 MPa), shear-thinning behavior (decreasing friction resistance), and unique biological properties. SF is the major component of silk (~75%) and encompasses three subunits, namely, the heavy chain, light chain, and P25 protein. SF is obtained via the degumming (removal of sericin, the outer glue-like coating material) of silkworm cocoons, *Bombyx mori*. Nevertheless, an aqueous solution of SF is difficult to cure and sometimes poses biocompatibility concerns due to sericin remnants. SF is approved by the FDA as SeriScaffold, a silk scaffold for soft tissue repair, and is widely used in textile, food, cosmetic, and tissue engineering applications.

SF hydrogels can be prepared using physical or cross-linking methods. Physical factors (pH, temperature, solvents, cations, and vortexing) induce a conformational transition from a random-coil structure to a β-sheet by the association of the hydrophobic block structure through hydrogen bonding and trigger gel formation. Chemical cross-linking can be mediated by photopolymerization or the use of chemical/enzymatic cross-linking agents [30,31,32]. Because of its higher mechanical integrity, Yodmuang et al. reported load-bearing scaffolds fabricated from silk-microfiber-reinforced silk hydrogel, where structural integrity was maintained in vivo for up to 42 days and the scaffolds mimicked the structure and function of native cartilage [33]. Recent advances have driven the development of composite scaffolds such as agarose/SF blended hydrogels [34] and photopolymerized maleilated chitosan/methacrylated SF [35] to better simulate the ECM matrix of cartilage and for superior injectability, adhesion, self-healing, and bioactive properties.

##### Others

In recent years, researchers have explored other protein polymeric materials such as sericin and elastin for hydrogel preparation. Sericin, a protein extracted from raw silk, consists of 17–18 amino acids and exhibits properties similar to those of SF in terms of hydrophilicity, biocompatibility, biodegradability, and non-immunogenicity [36]. It also possesses photoluminescent properties that enable the detection and imaging of a subcutaneously implanted double network (DN) hydrogel in vivo. However, it has shown technical limitations of degradation during extraction from silk. Therefore, hybrid sericin hydrogels such as sericin/HA/chondroitin sulfate [37], sericin/alginate [38], and sericin/gelatin/PVA [39] have been developed to treat arthritis.

Another protein polymer, elastin, is a highly cross-linked polymeric protein that is elastic and insoluble in nature [40]. Elastin molecules can be easily modified and cross-linked by covalent bonds. Therefore, elastin-based biomaterials such as elastin-like recombinamers (ELRs) have been developed for the preparation of injectable hydrogels encapsulated with mesenchymal stromal cells. The fabricated ELR-based bioactive hydrogel demonstrated excellent regeneration of hyaline cartilage and bone in a subchondral defect model of New Zealand rabbits [41,42].

#### 2.1.2. Polysaccharide-Based Hydrogels

**Figure 3:**
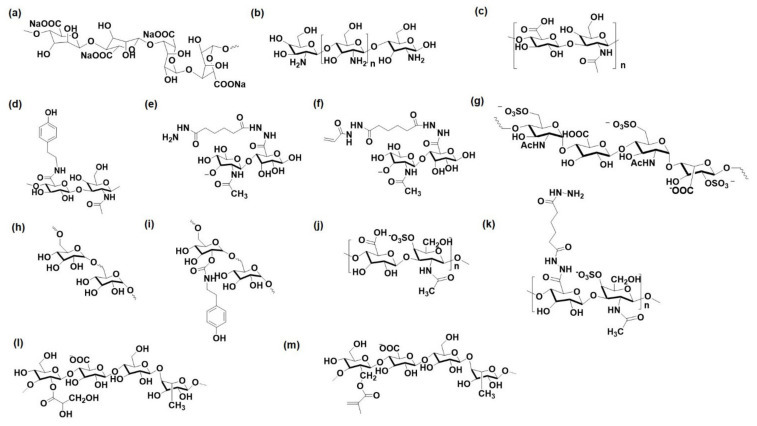
Chemical structures of polysaccharide polymers: (**a**) alginate (sodium salt), (**b**) chitosan, (**c**) hyaluronic acid (HA) and their modifications such as (**d**) HA–tyramine, (**e**) HA–adipic acid dihydrazide, (**f**) HA–acrylate, (**g**) heparin, (**h**) dextran and its (**i**) tyramine conjugate, (**j**) chondroitin sulfate and its modification, (**k**) chondroitin sulfate–hydrazide, and (**l**) gellan gum and its (**m**) methacrylate derivative.

Polysaccharide materials are appealing for hydrogel preparation because they possess characteristics similar to those of the natural cartilage ECM. Moreover, they have demonstrated excellent biocompatibility, biodegradability, non-toxicity, and high affinity toward biological molecules, and may regulate signaling pathways, leading to chondrogenesis. The polysaccharides possess several hydroxyl and carboxyl groups in their structure, allowing polymer modifications with methacrylate, acrylate, and aldehyde moieties. The chemical structures of the polysaccharides and their modified derivatives are shown in Figure 3. In this section, different types of polysaccharide materials are discussed along with the recent advancements in improving their drug/cell-loaded hydrogel characteristics (gelation and mechanical properties).

##### Alginate

Alginate is a naturally occurring anionic polysaccharide extracted from the cell walls of brown algae (Phaeophyceae). It consists of β-D mannuronic acid and α-L-glucuronic acid in different arrangements. Alginate is favorable for hydrogel preparation because it exhibits biocompatibility, non-immunogenicity, non-toxicity, and cost-effectiveness. Moreover, alginate is a FDA-approved polymer that is commercially available as 3D cell culture matrix (AlgiMatrix^TM^, QuickGel^TM^) and in wound dressings (NuGel^TM^). Divalent cations such as calcium ions (Ca^2+^) are often used to prepare hydrogels, where replacement of the Na^+^ ions of G-blocks with Ca^2+^ ions and the bending of glucuronic groups occur, forming an egg-box structure. However, ionic cross-linked alginate hydrogels exhibit insufficient mechanical strength, slow biodegradation, and poor cell adhesion, limiting their use in clinical applications [14,43].

To improve the mechanical strength and gelation properties of alginate gels, modified hybrid alginate was reinforced by the addition of a second polymeric material such as gelatin, HA, or 2-hydroxyethyl methacrylate. Balakrishnan et al. fabricated hydrogels by cross-linking the oxidized alginate with gelatin in the presence of borax, and reported a faster gelation time of 20 s and maintenance of chondrocyte viability and proliferation, as observed with increased expression of type II collagen, aggrecan, and enhanced glycosaminoglycan deposition [44].

##### Chitosan

Chitosan is a cationic linear polysaccharide extracted from the shells of shrimp and crabs by the deacetylation of natural chitin. Chitosan comprises D-glucosamine and N-acetyl glucosamine units and exhibits antimicrobial and bio-adhesive properties. Moreover, it has been approved by the FDA as HemCon^®^ for controlling hemorrhage conditions. Favorable structural, biocompatible, biodegradable, hydrophilic, and sterilizable properties make it an attractive hydrogel scaffold matrix. Chitosan is enzymatically degradable with a degree of deacetylation ranging from 15% to 85% [45].

Considering the weak mechanical and elastic properties of chitosan, hybrid chitosan-gelatin scaffolds [46], chitosan–HA [11] and supramolecular hydrogels [47] have been developed. The chitosan–gelatin hydrogel displayed a compression modulus of 47.9–72.5 kPa, which was 9–12 times higher than that of the pure chitosan scaffolds [46]. Taking advantage of the mechanical properties of the chitosan–gelatin scaffold, Han et al. designed a three-layered chitosan–gelatin hydrogel prepared by the photo-cross-linking of GelMA and carboxymethyl chitosan that mimicked the superficial, transitional, and deep zones of cartilage, exhibiting a porous structure (porosity of more than 80%) and higher compressive strengths of 65 ± 54, 565 ± 50, and 993 ± 108 kPa, respectively [47]. Recently, Mou et al. developed a supramolecular chitosan hydrogel via the cross-linking of N-carboxyethyl chitosan and adipic acid dihydrazide (ADH) with HA–aldehyde (through acylhydrazone and imine bond formation) [48]. The supramolecular hydrogel displayed a fast gelation time of 20 s, syringeability through a 26-gauge needle, long retention time up to 28 days with slow degradation, and self-healing properties. Furthermore, intra-articular injection of hydrogel decreased the expression of inflammatory cytokines and alleviated cartilage degeneration and synovial inflammation in a monosodium iodoacetate (MIA)-induced OA rat model [48].

##### Hyaluronic Acid

Hyaluronic acid (HA), also known as hyaluronan or hyaluronate, is a clinically accepted biocompatible, biodegradable material that possesses biological activities and activates many signaling pathways, so is widely used in bone regeneration. It is an anionic non-sulfated linear glycosaminoglycan comprising repeating units of D-glucuronic acid and N-acetylglucosamine, linked by β-1,4 and β-1,3-glycosidic bonds [49]. In aqueous solution, HA adopts a random-coil structure and forms a stiff, viscous, gelatin-like solution that displays high viscoelastic properties. HA hydrogels have reported a safe record of clinical use and have also received FDA approval for use as space fillers and to provide visco-supplementation [50].

Nevertheless, HA hydrogels exhibit weak mechanical properties and faster degradation in physiological milieu and are thus unable to maintain their structural integrity for a longer period of time [51,52]. The physiological properties of HA can be easily altered by cross-linking, through chemical modifications, or conjugation with other gelling polymers. Earlier, Elmorsy et al. developed a cross-linked high molecular weight HA hydrogel (6 × 10^6^ Da) and enzyme-linked HA-tyramine (HA-tyr) hydrogel to improve joint viscoelasticity and lubrication in both OA and RA [53]. Chang et al. explored HA hydrogels embedded with human umbilical cord mesenchymal stem cells (HUCMSCs). The HA hydrogel was able to conserve the morphology and proliferation of HUCMSCs in vitro. Further hydrogel scaffolds supported the significant gross and histological improvement in hyaline cartilage regeneration compared to those in the control knee in porcine OA models in vivo [54].

HA hydrogels have also been investigated for efficient drug encapsulation and potentiation of the therapeutic effects of anti-arthritic agents such as gapmer oligonucleotides [55], nanoiguratimod (NanoIGUR) [56], and infliximab (IFX) [57]. Cai et al. proposed a chemically cross-linked HA/chitosan hydrogel to encapsulate gapmer oligonucleotides to silence the cyclooxygenase-2 (*COX-2*) gene. The hydrogels loaded with the gapmer oligonucleotides displayed injectable properties and CD44-mediated target binding to chondrocytes with controlled gapmer release, and presented effective *COX-2* gene silencing ability over 14 days [55]. In another study, acrylate-modified HA was cross-linked with polyethylene glycol (PEG) (thiol)_2_ via Michael addition to synthesize a hybrid PEG–HA hydrogel, and iguratimod (IGUR) was loaded. The IGUR-loaded hydrogel demonstrated sustained drug release, improved pharmacokinetic profile, and reduced levels of inflammatory cytokines in vivo [56]. Overall, HA hydrogel composite scaffolds not only functioned as drug delivery carriers, but also served as tissue engineering scaffolds. HA-based hydrogels have shown immense therapeutic potential with excellent biocompatibility and biodegradable characteristics in arthritis therapies.

##### Heparin

Heparin, a negatively charged, highly sulfated linear glycosaminoglycan, comprises repeating units of 1,4-linked uronic acids and D-N-acetyl glucosamine and D-di-N-6-sulfate glucosamine [58]. Due to its negative charge, heparin interacts with various proteins associated with osteoblast cell adhesion (such as fibronectin and vitronectin) and osteogenic differentiation (bone morphogenic proteins such as BMP-2 and pleiotropin), thereby enhancing the activities of bioactive proteins (growth factors) and inducing osteogenesis, adipogenesis, and angiogenesis [59].

Heparin-containing gels have widely been used to deliver chondrocytes/MSCs and control the release of growth factors. To encapsulate cells and attain the controlled release of growth factors in arthritis treatment, various copolymers/copolymerization strategies have been attempted such as heparin copolymerized with PEG [60], alginate gels containing heparin [61], heparin-modified PEG star copolymer [62], heparin cross-linked with thiol-modified hyaluronan (HA-SH), or chondroitin sulfate with PEG diacrylate (PEGDA) [60]. These results show that heparin-integrated hydrogel networks may have promising potential for cartilage regeneration [63].

##### Dextran

Among the natural polysaccharide polymers, researchers have also explored the use of dextran in formulating hydrogels loaded with cells due to its hydrophilic and nontoxic nature, but it also revealed poor mechanical properties, as the dextran hydrogel was not able to support the chondrocyte load, demonstrating faster deformation and degradation. Dextran is a bacterial homopolysaccharide composed of α-1,6-linked d-glucopyranose and is excreted through the kidneys. The availability of various functional groups in the dextran structure led to the development of cross-linked dextran with favorable ECM conditions, biocompatibility, and improved mechanical properties [64].

Previously, dextran hydrogels were prepared by the radical polymerization of dextran methacrylate or maleic acid derivatives [65]. As an alternative to the photo-cross-linking method, dextran hydrogels based on dextran-tyramine (dex-tyr) conjugates were fabricated under physiological conditions by adding different concentrations of HRP and H_2_O_2_ to CTE. The dex-tyr conjugate showed a fast gelation time ranging from 5 s to 9 min, and high elasticity with G’ values ranging from 3 to 41 kPa [66]. Furthermore, the dex-tyr hydrogel showed efficient encapsulation of bovine chondrocytes and maintained cell viability, proliferation, and matrix production [67]. Recently, chemically cross-linked dextran-(PEG) [68], dextran-gelatin hydrogel [69], and biorthogonal dextran hydrogel [70,71] have been developed for enhanced chondrogenesis and cartilage regeneration. The biorthogonal dextran hydrogel showed the advantages of a catalyst-free cross-linking reaction of the dibenzocyclooctyne–dextran conjugate and azide–dextran conjugate under physiological conditions. Moreover, the dextran hydrogel led to better cartilage regeneration within six weeks compared to that in the positive control group (treated with agarose gel) with upregulation of chondrogenesis-related genes. This work highlighted the clinical feasibility of biorthogonal hydrogels for stem cell-mediated cartilage generation [70].

##### Chondroitin Sulfate

Chondroitin sulfate is a natural anionic linear polysaccharide composed of alternating units of β-1,3-glucuronic acid and β-1,3 N-acetylgalactosamine. It is abundantly present in the ECM of tissues such as cartilage, and exhibits cell adhesion and proliferation potential. To enhance the beneficial effects of cartilage repair with chondroitin sulfate, cross-linked polymers such as chondroitin sulfate/collagen [72], chondroitin sulfate/oxidized pullulan (oxPL) [73], and chondroitin sulfate (CS-SH)/hyperbranched PEG copolymer (HB-PEG) [74] have been developed. The chondroitin sulfate-adipic dihydrazide (CS-ADH)/oxPL hydrogel provided the merits of Schiff’s base reaction-mediated hydrogel preparation under mild conditions, showing a faster gelation time of 25 ± 4 s with a high swelling ratio of 26.7–67.1, compressive strength of 65 kPa, and a higher resistance to degradation with a weight remaining ratio of 42 ± 3% after 42 days of incubation. Moreover, in vitro studies with cell-laden hydrogel systems showed enhanced chondrogenesis with high expression of aggrecan, SOX9, and major ECM proteins (i.e., type I, II, and X collagen) [73].

Recently, Li et al. reported a click-enabled hydrogel, thiol-functionalized chondroitin sulfate (CS-SH) with multifunctional hyperbranched PEG copolymer (HB-PEG) to overcome the limitations (such as faster degradation and poor mechanical properties) associated with chondroitin sulfate. Thus, the developed CS-SH/HB-PEG hydrogel exhibited gelation within 3 min, with a G′ of 3–4 kPa and an ideal G′ range for stem cell chondrogenesis and induced in vitro chondrogenesis of adipose-derived MSCs with an 18-fold increase in the expression of the collagen type II gene. Thus, tunable biochemical and mechanical properties with combination polymer approaches present a strategy for enhancing cartilage regeneration with immense potential as drug delivery technologies [74].

##### Gellan Gum

Gellan gum (GG) is an anionic microbial polysaccharide produced by a non-pathogenic strain of *Sphingomonas elodea* [75]. GG is a nontoxic, biocompatible, and biodegradable polymer that shows excellent gelation properties. However, physically cross-linked GG hydrogels are less stable under physiological conditions and demonstrate limited cell infiltration and angiogenesis in vivo. [76]. Furthermore, GG also showed low aqueous solubility; therefore, GG methacrylate (GGMA) was synthesized, which showed high aqueous solubility and ease of preparation of the 1–2% aqueous solution. Moreover, to improve the stability and toughness properties of the hydrogel, modified GG hydrogels such as the GGMA/GelMA DN hydrogel embedded with cells [77], GG-tyr encapsulating betamethasone and anti-TNFα monoclonal antibody therapies (anti-TNF-α mAb) [78,79] and a graphene oxide-doped bilayered hydrogel consisting of GG and PEG diacrylate (PEGDA) were developed, which improved the lubrication properties and articular cartilage regeneration [80].

Initially, the DN hydrogel was prepared via the photo-cross-linking of methacrylated gelatin and GG derivatives, where it exhibited high mechanical strength with a high failure stress value of 6.9 ± 1.0 MPa and efficiently encapsulated NIH-3T3 fibroblasts while maintaining cell viability [77]. To side-step the use of a photoinitiator, enzyme-mediated hydrogelation at physiological pH and room temperature conditions was attempted using GG-tyr and betamethasone [81]. The GG-tyr hydrogel loaded with betamethasone showed a short gelation time of 30 s, superior elastic properties, slow degradation, and drug release, with only 50–60% drug release after 21 days, and enhanced in vitro chondrocyte proliferation with efficient neutralization of TNF-α levels. These results suggest that GG-tyr not only serves as a drug carrier, but also acts as a lubricant and shock absorber, alleviating arthritic pain and disease progression [78].

#### 2.1.3. Natural Hybrid Protein-Polysaccharide-Based Hydrogel

In general, protein-based polymers have shown the advantages of biocompatibility, biodegradability, and non-immunogenicity, but their drawbacks include faster degradation as well as poor mechanical properties, except for SF or sericin. Polysaccharide-based polymers have demonstrated superior viscoelastic properties but displayed a lack of cell-adhesive properties. Thus, fabricating a hybrid protein–polysaccharide-based hydrogel system could be a facile approach in developing a hydrogel material with significantly improved or substantially different properties from that of the non-hybrid hydrogels. Some examples of hybrid protein–polysaccharide-based hydrogels developed for arthritis treatment include SF/gellan gum-tyr loaded with betamethasone [82], sericin/alginate laden with mouse myoblasts [38], GelMA/chitosan encapsulating BMSCs [83], gelatin/HA-tyr loaded with epigallocatechin-3-gallate), and articular chondrocytes [84].

Generally, SF, sericin, and gelatin were blended and cross-linked with polysaccharide hydrogels to design hybrid protein/polysaccharide hydrogels, imparting higher mechanical strength/stiffness, sustained release, slow degradation, swelling, and porous characteristics to the hydrogel. In hybrid SF/HA-tyr hydrogels, improved mechanical properties have been demonstrated with the blending of SF and HA

**Figure 4:**
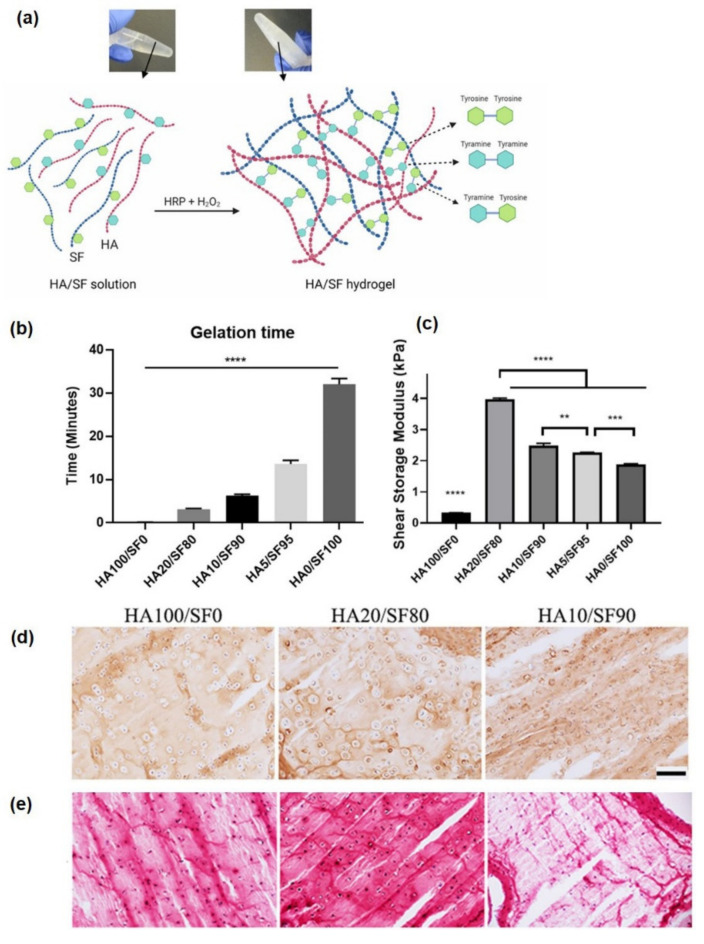
Schematic illustration of (**a**) the enzymatic cross-linked SF/HA-Tyr composite hydrogel, (**b**) the gelation time of the hydrogel was decreased with an increase in HA concentration, and (**c**) HA20/SF80 showed the maximum G′ values of 3.94 kPa among all the groups ** *p* <0.001 *** *p* < 0.0005 **** *p* < 0.0001. Immunohistochemical characterization revealed (**d**) maintenance of chondrocyte cell morphology on day 21 (Safranin-O staining) and (**e**) increased the accumulation of type II collagen (immunostaining). Adapted with permission from ref. [85], Copyright 2021 Elsevier.

The hybrid SF/HA-tyr hydrogel was prepared by cross-linking the mixed aqueous solutions of SF and HA-tyr via the addition of 10 U/mL of aqueous HRP solution and 0.01% H_2_O_2_. Figure 4 shows a schematic illustration of the SF/HA-tyr composite hydrogel with the sol–gel transition time with increasing HA concentration to SF and G′ values. The sol–gel transition time as determined by the vial inversion test showed a drop-in gelation time of pure SF hydrogel from 33 min to 13, 6, and 3 min with the addition of 5%, 10%, and 20% HA, respectively, to the SF solution. HA addition led to an increase in the degradation rate of the SF hydrogel and increased the swelling properties. Furthermore, HA20/SF80 showed the maximum G′ values of 3.94 kPa whereas the pure HA hydrogel displayed the lowest G′ values. Notably, the hybrid HA20/SF80 hydrogel maintained cell viability with increased expression of type II collagen and served as a promising mechanically reinforced hydrogel with cell immobilization with enhanced chondrogenic abilities [85].

### 2.2. Hydrogels Prepared Using Synthetic Material

Although hydrogels based on naturally derived materials exhibit negligible immunogenicity and biocompatibility and promote chondrocyte cell growth, proliferation, and phenotype preservation, their poor mechanical properties and unintended degradation generally restrict their clinical applications in RA and CTE. In contrast, hydrogels based on synthetic materials display controlled biodegradability and high mechanical and biochemical properties. Additionally, the degradation and mechanical properties of synthetic polymers can be modulated by changing the Flory interaction parameters (changing the monomer composition or its ratio) and cross-linking density. Synthetic polymers can be easily sterilized because of their resistance to degradation at high temperatures. However, synthetic polymers do not closely imitate the 3D microenvironment, and sometimes, degradation by-products of synthetic polymers may lead to secondary acute inflammation. Examples of synthetic polymers include poly(N-isopropylacrylamide) (PNIPAM), PEG-based copolymers such as poly(polyethylene glycol methacrylate) [poly(PEGMA)], PVA, and poloxamers. This section discusses the preparation methods, advantages, and arthritis treatment potential of commonly used synthetic polymer-based hydrogels and their modifications.

#### 2.2.1. Polyacrylamide-Based Hydrogels

Polyacrylamide-based hydrogels display thermoresponsive behavior with reversible swelling and deswelling, conforming to the precipitation-dissolution transition of the hydrogels. Poly(*N*-isopropylacrylamide) (PNIPAM or PNIPAAm) exhibits both hydrophobic and hydrophilic characteristics below and above the lower critical solution temperature (LCST) of 32 °C, facilitates chondrocytes/stem cell immobilization, and maintains cell viability and proliferation. To further enhance the chondrogenic ability of stem cells and promote cell-cell interactions, PNIPAAm is frequently cross-linked with other polymers such as chitosan-graft-PNIPAAm [86], PNIPAAm-PEG block copolymers [87], and hybrid PCL/PNIPAAm-PEG copolymers [88]. The hybrid PNIPAAm polymers displayed modulated LCST, swelling, thermal shrinking, and mechanical behavior. Chen et al. developed a thermoresponsive chitosan-graft-PNIPAAm injectable copolymer hydrogel with an LCST of 30 °C. Scanning electron microscopy (SEM) observations confirmed the porous-structured hydrogel with a pore size of 10–40 µm, which is suitable for immobilizing chondrocytes and meniscus cells [86]. In conjugation with PEG, a series of thermoresponsive PNIPAAm-PEG block copolymers with varying PNIPAAm contents and copolymer architectures, that is, linear, four-armed, and eight-armed configurations, was fabricated to encapsulate chondrocytes. The copolymers were synthesized by iniferter-based photopolymerization of dithiocarbamylated PEGs (DC-PEGs) under ultraviolet light exposure, and the LCST of the block copolymers varied from 31.4 to 34.0 °C. The LCST value decreased correspondingly with an increase in the block length of PNIPAAm owing to the formation of a branched configuration [87]. Similarly, Brunelle et al. developed an electrospun thermosensitive hybrid hydrogel scaffold consisting of PEG-PNIPAAm)/poly(ɛ-caprolactone) (PCL) and facilitated cell entrapment in a single-step cell seeding process and demonstrated mechanically stable behavior (compressive moduli of 35–40 kPa and increased viscoelastic properties) [88].

Furthermore, PNIPAAm-based hydrogels have also been investigated for delivering drugs such as celecoxib [89], siRNA [90] and suppressing nitric oxide (NO) levels, that is, NO-scavenging nanogel [91] with high safety and efficacy. Yeo et al. prepared an NO-responsive and -scavenging nanosized hydrogel (NO-Scv gel) by incorporating an NO-cleavable cross-linker (NOCCL) via a polymerization reaction. The NO-Scv gel showed a neutral surface charge and 5-fold more swelling than NO solubilized in water. In response to NO, the NO-Scv gel decomposes to benzotriazole and a carboxyl group, which are both responsible for NO scavenging.

**Figure 5:**
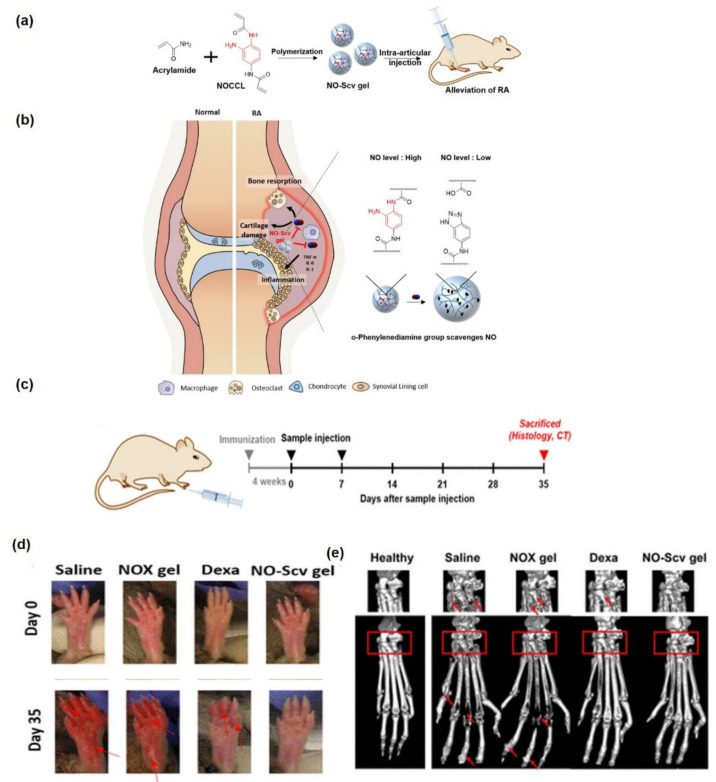
(**a**) Preparation of nitrous oxide (NO)-scavenging nanogel (NO-Scv) to alleviate rheumatoid arthritis. (**b**) NO-Scv nanogel prevents NO-mediated cartilage damage, inflammation, and bone deformation. (**c**) In vivo efficacy studies with NO-Scv nanogel in collagen-induced arthritis mouse model suggest (**d**) reduction in paw volume and swelling. (**e**) Monitoring of bone and joint morphology by computer tomography revealed clear boundary of bone and less bone erosion in both the ankles and fingers of mice as compared to those in the saline and NOx gel-treated animal group. Adapted with permission from ref. [91], Copyright 2019 American Chemical Society.

Figure 5 demonstrates the synthesis, mechanism, and therapeutic effects of NO-Scv gel in a collagen-induced arthritis (CIA) mouse model, where the NO-Scv treated animals showed no signs of redness, swelling, or decreased paw volume, while saline -and NOX-gel-treated animals showed increasing clinical scores. In this way, the biocompatible NO-Scv gel decreased the extent of inflammation by scavenging NO in vitro and suppressed the onset of arthritis in an in vivo mouse RA model, when compared with dexamethasone (dex) [91]. Notably, these studies presented a promising hybrid PNIPAAm-based hydrogel that show controlled release properties and hold great promise for osteogenic applications and as synthetic cartilage grafts. However, additional preclinical and clinical investigations are needed to examine the treatment efficacy of these acrylamide-based hydrogels.

#### 2.2.2. Polyethylene Glycol-Based Hydrogels

The synthetic polymer PEG has been widely studied and has attracted great attention for hydrogel fabrication for CTE and RA because of its excellent aqueous solubility, biocompatibility, biodegradability, and non-toxicity. It is an FDA-approved polymer; PEG-conjugated drugs are approved for safe use in humans [92]. The gelation and injectability of PEG-based hydrogels can be facilitated by in situ polymerization through photo-cross-linking, chemical cross-linking, and physical cross-linking.

Photo-polymerization-mediated cross-linking has been widely investigated by researchers in the synthesis of modified PEG derivatives such as oligo[poly(ethylene glycol) fumarate] (OPF) [93], RGD-modified PEG [94], PEG dimethacrylate (PEGDM) [95], and norbornene-terminated PEG macromers [96,97]. The RGD oligopeptide-modified PEG hydrogel has attracted great attention because of its enhanced cell adhesion, chondrogenesis, and osteogenesis properties.

**Figure 6:**
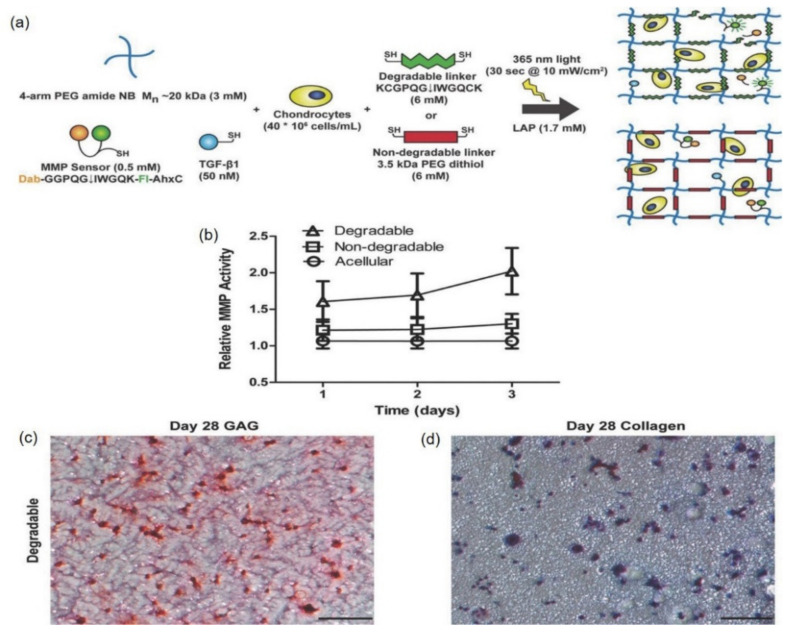
Schematic of MMP-degradable PEG hydrogel (**a**,**b**) prepared with a 4-arm PEG-norbornene network and MMP-degradable peptide sequence/or with non-degradable (3.5-kDa PEG dithiol) linker. The chondrocyte-laden degradable hydrogel maintained cell viability and significantly increased (**c**) GAG and (**d**) collagen deposition after 28 days of culturing. Adapted with permission from ref. [98], Copyright 2015 John Wiley and Sons.

Furthermore, norbornene-terminated PEG macromers were investigated to prepare a cartilage-specific-cellular degradable PEG hydrogel and prepared via the cross-linking of 4-arm (or 8-arm) PEG-norbornene macromers with matrix metalloproteinase (MMP)-degradable peptide [98,99]. The PEG-norbornene hydrogels were prepared by a step-wise photoinitiated polymerization reaction of 4-arm norbornene-terminated PEG with a PEG dithiol linker (non-degradable) or bis-cysteine collagenase-sensitive peptide cross-linker, allowing precise and temporal control over the polymerization reaction as well as facile encapsulation of TGF-β1 and cells. Figure 6 shows that the MMP-cleavable PEG scaffold also exhibited significantly increased gycosaminoglycans and collagen deposition after 28 days of culturing, while maintaining high levels of viability and generating a more diffuse matrix [98].

Along with photopolymerization, chemically cross-linked PEG hydrogels composed of vinyl sulfone-functionalized PEG (PEG-VS) and bis-cysteine MMP-sensitive peptide crosslinkers via Michael-type addition reactions were developed to support cellular viability and proliferation [100]. Furthermore, the Diels–Alder click reaction and covalent acrylhydrazone linkages were exploited to facilitate the intrinsic self-healing property of the hydrogel and monitor the on-off network switching, crosslink density, and adhesive properties [101]. In a recent study by Richardson et al., hydrazine covalent adaptable network (CAN) hydrogel was synthesized from 8-arm tripentaerythritol PEG macromers with reactive hydrazine, alkyl-aldehyde, and benzaldehyde end groups and showed excellent viscoelastic properties with a higher value of loss tangents with a decrease in angular frequency. Notably, chondrocyte biosynthesis results illustrated the higher matrix biosynthesis levels of cartilaginous sulfated GAGs and collagen over a one-month period, suggesting its practicality in cell-mediated remodeling and CTE applications in arthritis treatment [102].

#### 2.2.3. Poly(Vinyl Alcohol)

PVA hydrogels are widely investigated for CTE because of their non-toxicity, biocompatibility, hydrophilicity, tissue-like viscoelasticity, and ability to moderate modulus values of 1–5 MPa. Moreover, the very small pores in the PVA hydrogel structures facilitate the stowing of a large amount of water, imparting lubrication properties. PVA hydrogels are generally prepared by a physical cross-linking method employing cast drying (CD) or freeze–thaw (FT) cycling. In a comparative study by Oliveira et al., the tribomechanical properties of hydrogels designed by these two methods, CD and FT, were compared. The hydrogel prepared by the CD method displayed a dense structure, low swelling, and could withstand a higher load than the gels prepared by FT cycling [103].

However, slow biodegradation and poor cell adhesion properties have limited the use of PVA hydrogels for clinical cartilage tissue regeneration applications. Therefore, to enhance the bio-applicability of PVA hydrogels, hybrid PVA-based hydrogel composites combining PVA with PEG, PCL, poly(lactic-co-glycolic acid) (PLGA), alginate, chitosan, and bioglass have been extensively investigated [104]. In a similar context, Meng et al. fabricated a bioinspired PVA/graphene oxide-hydroxyapatite (GO-HA)nanocomposite hydrogel by an extrusion 3D printing method. The introduction of graphene oxide (GO) or GO-HA reduced the strength of intermolecular hydrogen bonds and improved dynamic viscosity, shear-thinning behavior, and 3D printability of the PVA hydrogel. Furthermore, printed biomimetic PVA/GO-HA nanocomposite hydrogels possess suitable compressive and tribological properties for cartilage tissue repair [105].

#### 2.2.4. Poloxamer

Poloxamers are widely used to prepare in situ hydrogel systems because of their thermosensitive properties. It is a nonionic triblock copolymer comprising the central hydrophobic part of poly(propylene oxide) (PPO) flanked by two hydrophilic chains of poly(ethylene oxide) (PEO). In response to physicochemical changes in pH, temperature, or ionic concentration, it self-assembles and forms a cross-linked hydrogel via micellar rearrangement. Madry et al. exploited the PEO–PPO–PEO poloxamer hydrogel Pluronic^®^ F127 to deliver a therapeutic recombinant adeno-associated virus (rAAV) vector overexpressing the chondrogenic *sox9* transcription factor and indicated that the rAAV-copolymer hydrogel augments microfractures and remarkably enhances cartilage repair with stabilization of the subchondral bone plate in the mini-pig model [106]. In another study, Zhang et al. prepared a glucosamine (GlcN)-encapsulated thermoresponsive hydrogel using poloxamer 407 and poloxamer 188 and investigated its therapeutic efficacy against OA by intra-articular administration. The hydrogel showed a sol–gel transition temperature (T_sol–gel_) at ~35 °C and a slow release of GlcN. The hydrogel-treated group (rabbit OA model) exhibited remarkably reduced swelling, reduced release of inflammatory factors, and improved repair efficacy compared to the non-treated group [107].

#### 2.2.5. Others

Similar to PEO/PPO-based hydrogels, polyglycerol sulfate (dPGS), poly(N-vinylcaprolactam) (PNVCL)-based, poly(ester)urethane (PEU)-based hydrogels, and block copolymers comprising PEG, PLGA, PCL, poly-D, and L-lactic acid have also attracted great interest in CTE due to their excellent biodegradability and tunable mechanical properties. For example, Schneider et al. prepared an in situ dPGS-based polyanionic hydrogel via a strain-promoted azide-alkyne cyclo-addition reaction to mimic a heparin-based 3D scaffold. The biorthogonal dPGS-PEG hydrogel demonstrated an elastic modulus varying from 1 to 5 kPa by varying the dPGS content and maintained higher cell viability with tissue formation [108]. PNVCL hydrogels were investigated because of their thermoresponsive in situ gelling properties, showing a LCST value similar to the physiological temperature [109]. Furthermore, to take advantage of the elastic and biodegradable properties of PEU, hydrogel scaffolds with different pore sizes of 200, 400, and 600 µm and Young’s moduli of 16 ± 3, 7 ± 1, 5.6 ± 0.5 MPa were developed [110]. These hydrogel scaffolds showed promising potential for use in the regeneration of articular cartilage defects.

Researchers have also investigated block copolymers for fabricating hydrogel composites because changing the polymer composition and concentration can alter the hydrogel network density. Furthermore, modification of the end-capping group may result in modulating release kinetics. Midwoud developed a celecoxib-loaded injectable hydrogel prepared from poly(ε-caprolactone-co-lactide) (PCLA)-b-poly(ethylene glycol)-b-poly(ε-caprolactone-co-lactide) (PCLA-PEG-PCLA), a triblock polymer containing an acetyl or a propyl end-cap, to locally treat OA. In vivo pharmacokinetic studies in rats following subcutaneous hydrogel injection showed an increase in the plasma half-life of celecoxib from 5 h to 10 days, supporting sustained-release gel formulation, with the propyl end-capped polymer hydrogel showing much more prolonged drug release than the acetyl end-capped polymer hydrogel [111].

### 2.3. Hydrogels Prepared Using Hybrid (Semi-Synthetic) Material

Hybrid (semi-synthetic) hydrogels, which include two or more natural/synthetic polymers, can integrate the advantages of their individual constituents. Therefore, semi-synthetic materials exhibit a diversity of physical and chemical properties to meet the preferred requirements of hydrogel networks such as structural integrity, biocompatibility, faster gelation, slow degradation, efficient loading of drugs and cells, and further potentiating curative effects in arthritis treatment. Remarkably, modified/hybrid composites showed improved gelling and mechanical properties, efficient encapsulation, and sustained release properties with long intra-articular retention compared to individual components, and demonstrated promising potential for use in arthritis treatment. Table 1 shows the improvement in hydrogel characteristics with respect to gelation time, temperature, and mechanical properties with the addition of the second hydrogel component, cross-linking mechanism, and therapeutic outcome in CTE and RA. This table may be useful for researchers to understand the effects of a particular polymer on the mechanical, rheological, and curative effects of hybrid-composite hydrogels.

## 3. Hydrogels as Depot Delivering System for the Treatment of Arthritis

Given the complex and multifactorial nature of arthritis, different biochemical pathways and tissue engineering approaches have been investigated to counteract this devastating musculoskeletal disease. They can be categorized as small molecule drugs (non-biologic DMARDs), immunotherapeutic agents (biologic DMARDs), and tissue engineering approaches via stem cell and/or chondrocyte delivery [123]. Conventional delivery of therapeutic agents resulted in a poor pharmacokinetic profile, leading to low therapeutic efficacy and side effects. Therefore, the novel targeted hydrogel-mediated delivery of non-biologic DMARDs, immunotherapies, and tissue engineering (bone regeneration and cartilage repair) has attracted more attention for the treatment of arthritis. In general, traditional DMARDs have shown a range of side effects such as gastrointestinal (GI) toxicity, skin reactions, and blood and hepatic toxicity, which vary from drug to drug [124]. Hydrogels circumvent the issue of toxicity via targeted delivery (i.e., intra-articular injection), which requires low drug doses and can eliminate the urgency of repetitive administration of anti-arthritic agents.

Additionally, designing hydrogels for targeting the molecular pathways that regulate the immune response has emerged as a specialized arthritis treatment approach to improve joint mobility. Cartilage and bone tissues are reported to have limited blood vasculature and low proliferation capacity [125]; the hydrogel scaffold serves as a template matrix and enables cell immobilization as well as the attachment of a greater number of surrounding cells for faster tissue regeneration. Due to their tunable physical properties, controllable degradability, and capability to protect labile drugs from degradation, hydrogels serve as promising delivery vehicles for treating arthritic disorders.

Hydrogel mediated controlled drug release varies according to hydrogel network degradation, swelling, mechanical deformation and drug–polymer interaction. In particular, diffusion is the most widely applied drug release strategy and dominated when the mesh size is larger than the drug molecule. However, there are difficulties in controlling burst release. Thereby, the key parameter regulating hydrogel mediated drug diffusion and release is the mesh size that varies according to the gelation method. Swelling or degradation leads to mesh size enlargement, allowing drugs to diffuse out from hydrogel. Swelling-controlled systems display slow drug release due to the slow diffusion of water. Thereby, to achieve faster release with swelling-controlled systems, various approaches such as altering the bulk structure of hydrogel, pH, or temperature sensitive systems have been developed. Hydrogel degradation is typically mediated by hydrolysis or enzyme action and differentially regulate bulk and surface erosion, whereas matrix deformation may be triggered by mechanical deformation or ultrasound and magnetic field-induced deformations. Stronger interactions between drugs and polymer chains is another approach to design hydrogel controlled drug release systems, providing drug protection and immobilization, where highly stable cleavable covalent linkages, electrostatic interactions, or hydrophobic domain can be introduced between the drug and polymer [126,127].

Different types of hydrogels for encapsulating drugs, immunotherapeutic agents, and cells showing improved pharmacokinetic and pharmacodynamic profiles are discussed in this section.

### 3.1. Hydrogels Delivering Small Molecule Drugs

Small molecule drugs including NSAIDs, glucocorticosteroids, immunosuppressants, and DMARDs are in use to relieve pain and reduce inflammation in arthritis. NSAIDs such as ibuprofen, celecoxib, naproxen, and valdecoxib are fast-acting anti-inflammatory agents that are often adopted as the first-line management of arthritis [123]. However, prolonged systemic administration of NSAIDs at higher doses is associated with an increased risk of various cardiovascular events, gastrointestinal toxicity, and ulcers [128]. Therefore, Cokelaere et al. developed a celecoxib-loaded thermoresponsive hydrogel from a synthetic triblock copolymer comprising propyl-capped PCLA–PEG–PCLA and evaluated its efficacy in a lipopolysaccharide (LPS) challenge equine synovitis model. Both the high-dose and low-dose celecoxib-loaded hydrogel showed sustained and controlled intra-articular drug release for up to 30 days, without any adverse effects. Furthermore, the high-dose celecoxib hydrogel significantly inhibited white blood cell concentration 8 h after the LPS challenge [129]. Glucocorticoids are another effective alternative, but systemic administration of corticosteroids is only indicated for a short period of time and at lower doses due to reported toxic effects [130]. A recent study reported the development of GEL–MAN hydrogels fabricated with benzoxaborole polymers for the topical delivery of triamcinolone acetonide (TCA) at the joint cavity. The GEL–MAN hydrogel was advantageous because it was degraded by the H_2_O_2_ released by the arthritic joint and consequently controlled TCA release. Intra-articular injection of TCA-loaded GEL-MAN in an OA rat model resulted in improved treatment outcomes with significantly reduced joint tissue damage compared to those in the no-treatment group [131].

Similar to other small molecule drugs, systemic administration of MTX is often discontinued due to pneumonitis, liver toxicity, and bone marrow deterioration. Alopecia (particularly in women) and chest problems are major concerns with MTX use [124]. To circumvent MTX-associated toxicities, a poloxamer-based thermosensitive hydrogel containing MTX-loaded polyelectrolyte complexes was developed. Thus, the developed poloxamer hydrogel demonstrated prolonged MTX release, and further in vivo studies in RA rats via intra-articular injection resulted in recovered cartilage damage and decreased allodynia [132]. In this way, hydrogels provided enhanced therapeutic efficacy at low doses and frequencies while circumventing drug-associated toxicity issues to provide symptomatic relief in arthritis.

### 3.2. Hydrogels Delivering Immunotherapeutic Agents

In recent years, improved understanding of the molecular pathology underlying arthritis has led to the emergence of a number of immunotherapeutic agents that are being investigated to modulate the levels of arthritis-associated inflammatory cytokines, immune cell activity, and inhibiting enzymes [133]. These are mainly oligonucleotide-based therapeutics such as antisense oligonucleotides (ASOs) targeting specific mRNAs, cytokine inhibitors for inhibiting osteoclast activation, and bone resorption such as TNF-α inhibitors (IFX, etanercept, certolizumab pegol, etc.) and nuclear factor-κB inhibitors such as IGUR and interleukin inhibitors. These therapeutics decrease the release of inflammatory cytokines at the joint synovium and systemic circulation [133,134,135,136].

Oligonucleotide-based therapeutics for arthritis have shown remarkable progress over the last few years with the potential to inhibit the synthesis of specific proteins within cells such as chondrocytes, fibroblasts, and osteocytes and further lower the levels of inflammatory cytokines or catabolic enzymes in blood or synovial fluid [137,138]. However, ASO-based therapeutics face challenges in crossing the formidable biological barrier of articular cartilage because of their size and anionic charge. Biomaterial carriers such as hydrogels have emerged as delivery vehicles, providing sustained release of ASOs at the joint site for a long time. In such an attempt, Garcia et al. reported a fibrin–HA hydrogel to target matrix degrading ADAMTS5 (a disintegrin and metalloproteinase) via encapsulating gapmers (locked nucleic acid-modified antisense oligonucleotides, LNA-ASO). This hydrogel-based platform displayed a 14-day sustained release of the incorporated LNA–ASOs and an efficient uptake by primary human osteoarthritis chondrocytes [26]. In summary, HA-based delivery reinforces the dual ability to deliver chondrocytes along with gapmers to increase the bioavailability of therapeutics in the synovial cavity and modulate catabolic gene expression and cartilage repair.

Cytokine-targeting therapies such as anti-TNF-α mAb (for e.g., IFX) have shown a decrease in TNF-α production by activated synoviocytes and articular chondrocytes, which are responsible for matrix degradation and disease progression. However, only a limited number of patients with arthritis have responded to therapy; moreover, there is a heightened risk of cardiovascular diseases in patients with RA [139]. To improve the bioavailability of anti-TNF-α mAb and reduce its toxic effects, Chen et al. fabricated a thermoresponsive injectable hydrogel by mixing Pluronic^®^ F127 and HA with poly(γ-glutamic acid) and investigated the cartilage protection effects of the local delivery of IFX in a rabbit model of RA. Release studies recorded a sustained IFX release for over 28 days from the hydrogel, and in vivo efficacy studies displayed relatively smooth articular cartilage surface and reduced synovial inflammation compared to damaged cartilage features observed in the control group [140]. Recently, an improved hydrogel system based on anti-TNF-α mAb-chondroitin sulfate/PAMAM dendrimer NPs loaded with Ty-GG and Ty-GG/SF hydrogels was developed by Oliviera et al. for TNF-α capture. In vitro studies showed that the anti-TNF-α mAb/dendrimer NP-loaded hydrogels showed sustained release of mAb over 21 days with the maintenance of high therapeutic effect over time in THP-1 cell-based assays.

Likewise, NF-κB inhibitors such as IGUR, have also shown effectiveness in mitigating arthritic symptoms, however, their clinical applications are limited because of GI toxicities. To improve bioavailability and overcome toxicity issues, NanoIGUR was loaded into the HA-acrylate/dithiol PEG hydrogel. The NanoIGUR-loaded semi-synthetic hydrogel improved the bioavailability of IGUR, displayed sustained release, and minimized the GI side effects with a higher area under the curve (AUC) value of 150 µg/mL, lower C_max_, and delayed T_max_ compared to those values in the free IGUR solution. Moreover, treatment with the NanoIGUR-loaded hydrogel in a CIA rat model led to decreased expression levels of inflammatory cytokines in the serum as well as synovial tissue homogenate and decreased arthritis index and pathological score, suggesting the clinical applicability of the NanoIGUR-loaded hydrogel [56]. As a result, effective targeted delivery strategies via hydrogels successfully transported the therapeutic cargo to the joint site and presented high therapeutic efficacy with minimal side effects by maintaining immunotherapeutic concentration at the joint site over a long period of time.

### 3.3. Hydrogels Delivering Cells

Cell-based therapies with chondrocytes and MSCs have shown the ability to repair damaged joint surfaces in arthritis with a functional tissue capable of withstanding the stresses and strains of joint loading. Cell-based therapies involving autologous chondrocyte implantation require a sufficiently large number of cells, obtained after multiple passages and expansion process of the monolayer culture, which may sometimes lead to dedifferentiation of chondrocytes, which is unfavorable [141]. In addition to chondrocytes, cell-based therapies via MSCs are advantageous because MSCs are multi-potent and easy to isolate [142]. Despite their potential advantages, cell-based therapies mostly suffer from the drawback of a lack of structural support.

Therefore, to efficiently regenerate load-bearing tissue such as cartilage, an initial structural support showing sufficient mechanical toughness such as a biomimetic hydrogel scaffold/cell-laden hydrogel scaffold has been developed. Using advanced techniques, cell-encapsulated hydrogels can be fabricated with personalized geometries and compositions and they demonstrate immense potential in maintaining cell viability and providing an optimal niche to promote chondrogenesis. In recent years, various modifications of hydrogel systems have been investigated for loading cells and cartilage regeneration [143]. Additionally, cell-laden hydrogels may be combined with anti-inflammatory drugs and biological cues (i.e., growth factors, peptides, and siRNAs) to maintain the chondrogenic phenotype, enhance cell–matrix interaction, generate hyaline neotissues in cartilage focal defects, and alleviate inflammation [97,102,144,145].

In particular, hybrid hydrogels comprising both natural as well as synthetic components such as glycol chitosan/benzaldehyde-functionalized PEG [112], HAMA/pluronic nano-micelli [113], alginate-poloxamer/SF [115], PEG/SF [119], PEG/gelatin-norbornene [145] as well as MMP-sensitive PEG hydrogels [97] and hydrazone CANs [102] have been developed. These hybrid hydrogels facilitate long-lasting viability, maintain cellular phenotype, and promote proliferation with the generation of cartilage ECM including aggrecan, decorin, and collagen type II. For example, Neethu et al. encapsulated rabbit chondrocytes in a chitosan/HA dialdehyde hydrogel and implanted them in rabbit knee joints with osteochondral defect. After 12 weeks of implantation, animals treated with cell-laden hydrogels regenerated cartilage similar to the surrounding native cartilage [146]. The effect of the stiffness of chitosan/HA dialdehyde gel on the viability and growth of encapsulated chondrocytes was investigated by Thomas et al. Chondrocyte culture studies (28 days) carried out with hydrogels of varying stiffness demonstrated that the stiff gels helped to maintain the spherical phenotype and presented a uniform cell distribution and formed spherical aggregates with enhanced ECM production. In contrast, the less stiff gels showed good cell viability during the initial seven days of culture. These results indicate that the stiffness or elasticity of the hydrogel substrate should be properly tuned to match the native ECM elasticity while designing an in vitro microenvironment for cartilage regeneration [147]. Recently, Richardson et al. designed a viscoelastic PEG-based hydrazone CAN, which was seen as a dynamic compression bioreactor for chondrocyte encapsulation. The developed hydrazone CAN facilitated cartilage ECM synthesis under an intermediate level of viscoelastic adaptability and induced the highest matrix biosynthesis levels of cartilaginous sulfated GAGs and an average of 31 ± 3 µg of collagen per day over one month in chondrocyte culture under dynamic compressive loading [102].

Similarly, a PEG/collagen mimetic peptide (PEG/CMP) hybrid hydrogel was developed as a scaffold for the encapsulation, proliferation, and differentiation of human MSCs (hMSCs) into neocartilage/chondrocytes. The results demonstrated that PEG/CMP hydrogels promoted chondrogenesis of hMSCs and enhanced the secretion of cartilage-specific ECM. Furthermore, chondrogenesis was found to be affected by matrix elasticity, where the soft matrix induced a greater degree of chondrogenic differentiation and the stiff matrix showed limited chondrogenic differentiation, likely due to restricted mass transport [148]. A recent study showed the development and characterization of a facile photo-cross-linkable PEG/norbornene gelatin hydrogel system for in situ transfection of MSCs along with mechanosensitive miRNAs (miR-100-5p/miR-143-3p). Delivery of both MSCs and miRNAs via hydrogels led to superior mineralization and osteogenic gene expression in vitro [145]. Given the positive outcomes of cell-laden hydrogel scaffolds for regeneration and replacement of cartilage tissues, hydrogels hold future applicability in CTE. However, the hydrogel characteristics require improvement for enhancing tissue regeneration, and further preclinical studies are warranted to validate their efficacy and feasibility in clinical translation.

## 4. Recent Advances in Hydrogels

Despite the advantages and potential applications of hydrogels, they also suffer from the limitation of non-uniformity in the dispersion of hydrophobic drugs and decreased joint residence time. In an effort to maintain a drug depot at the joint site and to increase the payload as well as uniform dispersion of therapeutic agents, nano/microcomposite integrated hydrogels have been designed with greater efficacy. Current strategies are directed at the development of hydrogels loaded with liposomes and particulate systems (nanoparticles (NPs) and microparticles (MPs)) to achieve high therapeutic efficacy at low doses and to minimize side effects [3,149]. Table 2 shows the development of the liposome-, microparticle/microsphere-, nanoparticle/nanocapsule/nanomicelle-loaded hydrogel composite for delivering drugs and cells to joint sites with improved curative effects.

### 4.1. Liposome-Loaded Hydrogel System

Lipid carriers such as liposomes, niosomes, and aspasomes containing biologically active molecules have attracted much attention because of their self-assembling properties, biocompatibility, and biodegradability. Niosomes are lipid bilayer vesicles formulated using nonionic surfactants, while aspasomes are lipid vesicles containing ascorbic acid and its derivative as a component of the lipid bilayer. Liposomes can encapsulate both hydrophilic and hydrophobic compounds in the inner aqueous compartment and bilayer membrane structure, respectively. However, it is difficult to achieve sustained drug release from hydrophobic drugs using liposomes. Therefore, liposome-loaded hydrogels were fabricated, which may overcome the issues of burst drug release and instability posed by conventional liposomes.

In one such study, a triptolide (TP)-loaded liposome hydrogel (TP-LHP)-based transdermal delivery system was developed to improve the pharmacokinetics and pharmacodynamics of TP. TP exerts potent pharmacological effects against RA by acting as an anti-inflammatory and immunosuppressive agent; however, its clinical use is limited due to GI, liver, cardiac, hematopoietic, and urogenital toxicities [160]. Hence, microneedle-mediated transdermal delivery of TP-loaded liposome hydrogel was attempted, which assisted in bypassing the hepatic first-pass metabolism of TP, minimizing drug toxicity, and maintaining plasma drug levels. In brief, TP-loaded liposomes were initially prepared from egg lecithin and cholesterol (5:1) via a thin film hydration method and the TP-loaded liposomes were then loaded into the hydrogel, which was prepared by mixing phase A (a mixture of viscomate NP-700, glycine aluminum, polyvinylpyrrolidone K-90) and phase B (aqueous solution of tartaric acid). Thus, the developed TP-LHP reduced the serum cytokine levels of IL-1β and IL-6 and decreased the expression of fetal liver kinase-1, fetal liver tyrosine kinase, and hypoxia-inducible factor-1α in the synovium, eventually reducing joint swelling in CIA rats [161].

Correspondingly, MTX aspasomes containing ascorbic palmitate were developed and loaded into a carbopol gel for transdermal drug delivery in arthritis. Aspasomes were formulated from a mixture of dimyristoylphosphatidylcholine (DMPC), cholesterol, and ascorbic-6-palmitate (60:30:1) by the thin film hydration method and MTX was loaded. Carbopol 940 was used as a gelling agent. The hydrogel was characterized for viscosity, drug release, and therapeutic efficacy in an adjuvant-induced arthritis (AIA) rat model, where topical application of the MTX formulation demonstrated a positive outcome with reduction in cytokine levels, inflammation, cartilage damage, pannus formation, and inhibition of bone resorption compared to those parameters in the free MTX-treated arthritic rats [151].

### 4.2. Polymeric Particulate-Loaded Hydrogel Systems

In addition to lipid carrier-loaded hydrogels, researchers have also investigated microparticles (MP) and nanoparticle (NP)-loaded hydrogel systems to increase the joint residence time of therapeutic agents and circumvent toxicity issues. Encapsulating polymeric NPs/MPs in hydrogels has overcome the limitations associated with polymeric systems; for example, NPs smaller than 250 nm have a tendency to rapidly escape from the joint cavity, while MPs are rapidly phagocytosed by macrophages present in synovial linings, enhancing their therapeutic potential for arthritis treatment [162,163,164].

Kucukturkmen et al. developed an injectable intra-articular hydrogel formulation containing diclofenac sodium (DS)-loaded PLGA and PCL NPs. In brief, PLGA and PCL NPs containing DS were prepared by a modified water/oil/water (w_1_/o/w_2_) emulsion solvent evaporation technique and then loaded into a thermosensitive hydrogel, prepared from poloxamer 407 and chitosan. Hydrogels loaded with PLGA NPs containing DS demonstrated controlled drug release over 30 days, increased the terminal plasma half-life of DS (from to 1–2 h to days), and thus proposed a once a month intra-articular DS hydrogel administration, improving patient compliance [165].

**Figure 7:**
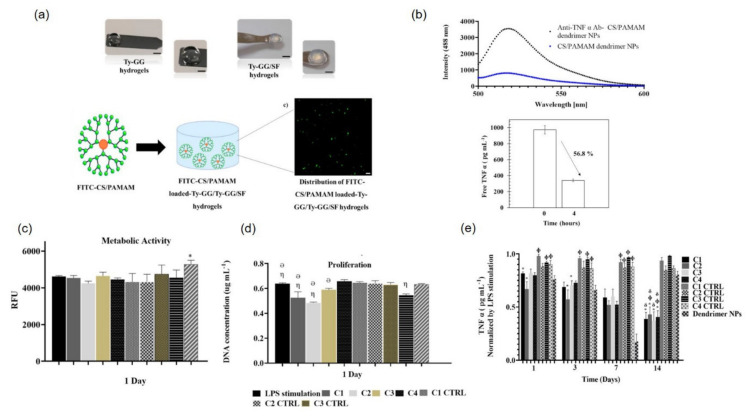
Anti-TNF α monoclonal antibody conjugated-chondroitin sulfate modified poly(amidoamine) dendrimer NP (Anti-TNF α mAb-CS/PAMAM dendrimer NP)-loaded GG-tyr and GG-tyr/SF hydrogel. (**a**) Representative images of GG-tyr and GG-tyr/SF hydrogel, where the fluorescence image shows a uniform distribution of NPs throughout the hydrogel. (**b**) Anti-TNF-α mAb conjugation to CS/PAMAM dendrimer NP. Effect of the hydrogel on THP-1 cell-based inflammation model, (**c**) metabolic activity, and (**d**) DNA concentration. (**e**) Measurement of the free TNF-α levels in cell culture media demonstrates that the anti-TNF α mAb-CS/PAMAM dendrimer NP-loaded hydrogel maintained cell viability, induced cell proliferation, and retained the capacity to neutralize TNF-α, even after 14 days. Adapted from [166] under the terms of the creative common attribution license, MDPI, 2021.

Recently, Oliviera et al. developed anti-TNF-α mAb-conjugated dendrimer NPs encapsulated in GG-tyr and GG-tyr/SF hydrogels in order to provide long-term anti-TNF-α mAb therapies for RA treatment. GG-tyr and GG-tyr/SF displayed advantages of the tuned hydrogel network showing fast gelation, high mechanical strength, resistance to degradation, and controlled drug release [78,82]. Chondroitin sulfate modified poly(amidoamine) (CS/PAMAM) dendrimer NPs aided in the conjugation of anti-TNF-α mAb. In brief, anti-TNF-α mAb-conjugated CS/PAMAM dendrimer NPs were synthesized via a carbodiimide chemistry reaction using a mixture of anti-TNF-α mAb, EDC (5:1), and CS/PAMAM dendrimer NPs. In the second step, GG-tyr/SF hydrogel was prepared by mixing an aqueous solution of GG-tyr (1% *w*/*v*) with SF (2% *w*/*v*), followed by the addition of HRP, H_2_O_2_, and NPs. Confocal microscopy with the FITC-labeled anti-TNF-α mAb-conjugated CS/PAMAM dendrimer NP-loaded hydrogel showed a uniform distribution of NPs throughout the hydrogel and release profile up to 21 days. The NP-loaded hydrogel retained the capacity to neutralize TNF-α, even on day 14 (Figure 7). In addition, dynamic in vitro studies using THP-1 cell-based inflammation with anti-TNF-α mAb-CS/PAMAM dendrimer NP-loaded hydrogel showed that the hydrogel maintained a high therapeutic concentration over a longer period and abruptly decreased the level of free TNF-α on day 14, thus demonstrating their promising therapeutic potential in arthritis treatment [79,166].

**Figure 8:**
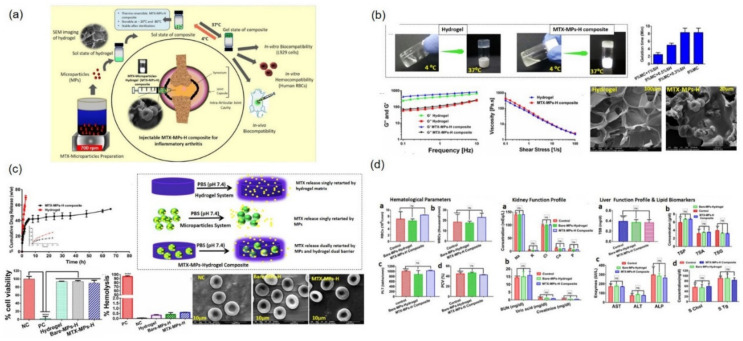
(**a**) Injectable methotrexate MP–hydrogel composite for anti-arthritic application. (**b**) T_sol–gel_, gelation time, viscoelastic properties, and morphology. (**c**) Hydrogel-mediated controlled MTX release. (**d**) In vivo biocompatibility studies. Adapted with permission from ref. [157], Copyright 2021 Elsevier.

Correspondingly, the MP-loaded hydrogel composite system was fabricated to deliver drugs and cells. Dhanka et al. developed a MTX–MP hydrogel composite to overcome the concerns of low water solubility of MTX and short in vivo half-life, requiring repetitive injections. The hybrid MP-hydrogel was fabricated with MTX-loaded alginate MPs (prepared by a water-in-oil emulsion and cross-linking with CaCl_2_) and a thermosensitive hydrogel (sodium hyaluronate blended with methylcellulose). SEM analysis of the injectable microparticle composite system showed that MPs were intact and irregularly shaped but uniformly embedded in the polymeric hydrogel. Investigation of the in vitro degradation behavior of the injectable gelling system showed 42% degradation within 33 days, and hemocompatibility was assessed via a hemolysis assay. In vivo injection and toxicity assessment by hematological, kidney function, liver function, and lipid profile assays demonstrated that there were no abnormalities such as redness or edema at the injection site, and there were no significant alterations in serum biochemical levels, indicating the safety of the developed MP–hydrogel composite system, as shown in Figure 8. Further curative studies in the AIA rat model demonstrated a significant decrease in the extent of edema and swelling in the arthritic paw [157]. Overall, these results strongly suggest that particulate–hydrogel composite systems are promising tools for controlled drug delivery and they increase the joint residence time of drugs while mitigating drug toxicities, thus warranting further exploration.

Furthermore, researchers have attempted to recreate inorganic mineral phases by the inclusion of bioresorbable ceramics such as tricalcium phosphate, nano-hydroxyapatite (nHAp), nanobioglass ceramics (nBGC), and wollastonite in hydrogels for bone tissue engineering, with the aim of achieving better mechanical performance with hydrogel composites [167]. Ceramic materials have already shown high apatite-forming ability and improved osteoblast proliferation, differentiation, and mineralization, but are brittle and present difficulties in loading bioactive molecules [168]. Among these materials, bioactive glass (BG) is most commonly used in regenerative medicine, tissue engineering, and dentistry because of its biocompatible, osteoconductive, and osteoinductive properties [169]. In a study by Liu et al., a DN hydrogel loaded with BG was designed to repair osteochondral defects and meet the mechanical strength requirement to support cartilage growth. The DN hydrogel was fabricated from a glycol chitosan (GC)/dibenzaldehyde PEO network and sodium alginate/CaCl_2_ to load BG and growth factor (TGF-β1). Full-thickness osteochondral defect model studies in New Zealand white rabbits with the DN hydrogel containing BG and TGF-β1 reported excellent ability of the hydrogel to reconstruct the subchondral bone, and proposed its potential clinical applications in arthritis treatment [170]. Similarly, Zhou et al. developed a hybrid gelatin/oxidized chondroitin sulfate hydrogel containing BG NPs for bone regeneration and showed high osteogenic differentiation of rat BMSCs in vitro and rat cranial defect restoration in vivo, presenting promising results. However, such composite hydrogel approaches require further investigation in AIA/CIA animal models for clinical translation [171].

## 5. Challenges and Future Perspectives

Hydrogels have offered attractive therapeutic options for arthritis by reducing drug toxicity and demonstrating enhanced efficacy at low-dose treatment compared to conventional dosage forms. Despite the many advantages, there are some issues when working with hydrogels. The key concern is the development of a 3-dimensional hydrogel structure showing improved mechanical integrity. The hydrogels displaying low tensile strength cannot be used for load-bearing applications as they may lead to premature dissolution or are unable to maintain the depot. Thus, hydrogels developed with hybrid semi-synthetic materials need to be explored. Further efficient entrapment of drugs within the hydrogel matrix, preventing burst release, and maintaining controlled release is another challenge, where the drug loading and drug homogeneity is regulated by hydrogel pore size. This limitation can be overcome by the preparation and processing of scaffolds with the applied nanotechnology and materials, where liposome-loaded, polymeric particulate loaded, and inorganic mineral phases can be introduced. Thus, the developed current hydrogel systems need to be investigated extensively in the future.

Furthermore, the lack of preliminary efficacy and safety studies in animal models has restricted hydrogel applications in clinical arthritis. The multifactorial complex nature of arthritis demands combination treatment approaches. Delivery of multiple therapeutic agents is required to achieve symptomatic relief, inhibit mediators of immunopathological response, and improve joint mobility via cartilage/bone tissue repair and regeneration. Delivering combination regimens with hydrogels has not been extensively explored. Only a few research reports have been published on hydrogel-mediated combination therapies. In one such report by Ghost et al., the beneficial effects of combination therapy of MTX with ascorbic acid via topical delivery of a MTX-optimized hydrogel were investigated. MTX-mediated side effects were reduced by decreasing H_2_O_2_ production with the combined use of the antioxidant ascorbic acid, and it offered a combination and a superior, non-invasive treatment option for RA [151]. However, there remains a need to establish a combination dosage regimen (dose and dosage frequency) in larger animals in order to extrapolate the results in humans.

Challenges also include studies correlating hydrogel characterization with a sustained release profile and further selection of animal species for in vivo efficacy studies. Generally, sustained release profiles are demonstrated by in vitro drug release studies conducted in PBS, which are not true representations of controlled release characteristics. Therefore, to establish a better in vitro–in vivo correlation (IVIVC), skin penetration properties should also be determined in addition to a sustained release profile. Furthermore, drug diffusion and the depot formation behavior of drugs varies among animal species, for example, the average cartilage thickness of common experimental animals such as mice, rats, and rabbits is within 700 µm, while the human cartilage is 1.5–2.0-mm thick; therefore, this should be taken into consideration when extrapolating results from animals to humans [172].

To evaluate the therapeutic efficacy of hydrogel delivery systems, animal disease models such as CIA/AIA (RA models) and MIA-induced OA rat model showing disease features similar to human RA and OA were used. Nonetheless, testing of hydrogel-based delivery systems in animal models has shown low predictability. Such issues have also been reported by researchers. In one such study, Lin et al. developed a microsphere/hydrogel system for a dual delivery of TGF-β3 and ghrelin to induce chondrogenic differentiation of hMSCs for cartilage repair and conducted cartilage repair experiments in a rat model. In vitro experiments have shown chondrogenic differentiation of stem cells; however, in an osteochondral defect rat model, the therapeutic results were not commendable, and the reason is that cartilage repair in rats does not represent the repair process in humans. Therefore, the authors proposed the use of miniature pigs to test cartilage repair as the joints of adult mini-pigs are upright, and their physiological and repair processes are similar to those of humans [173,174]. Therefore, the selection of both in vitro and in vivo animal models (small/large animals) should be taken into consideration when investigating hydrogel composite systems and for efficient clinical translation in humans.

Injectable hydrogel has become a major interest in arthritis treatment and some clinical trials are also undergoing where intra-articular injection of PVA hydrogel (NCT04693104), hydroxyethyl cellulose hydrogel (PROMGEL-OA, NCT04061733), polyacrylamide hydrogel (Aquamid, NCT03060421, and PAAG-OA, NCT04045431), polyacrylamide hydrogel with silver ions (i.e., NOLTREX ^TM,^ NCT03897686, and Argiform (NCT03897686), high molecular weight hyaluronan hydrogel (Hymovis, Fidia Farmaceutici NCT01372475, Phase III), non-cross-linked hyaluronic acid acrylamide (HYADD^TM^ 4, NCT02187549), a combination of human umbilical cord blood-derived mesenchymal stem cells, and hyaluronic acid hydrogel (CARTISTEM^®^, NCT01733186, Phase I/II) for the treatment of knee osteoarthritis are being investigated. Altogether, injectable hydrogels face challenges with regard to the loading of therapeutic agents, controlled release, mechanical strength, degradability, biocompatibility, hydrogel characterization, and therapeutic efficacy studies in vitro/in vivo and further scale-up approaches. The application of hydrogels and recently advanced hydrogel systems for improving arthritic condition remains a highly active area of research that needs to be evaluated in humans.

## 6. Conclusions

It is fortuitous that various investigations are ongoing on hydrogel-mediated targeted delivery of therapeutic drugs for the management of arthritis, but very few of them have recorded clinical therapeutic efficacy or received FDA approval. Hydrogels have emerged as potential therapeutic delivery platforms and regenerative medicine and filled the gaps with traditional therapies. Therapeutic-loaded hydrogels have demonstrated improved therapeutic outcomes (anti-inflammatory effects, reduced cytokine response, cartilage regeneration, and weight gain) at reduced drug doses. These hydrogels aid in reducing the frequency of intra-articular injection with non-biologic as well as biologic DMARDs and have better patient compliance. Despite the potential benefits and advantages of hydrogels, data related to preclinical safety and efficacy studies are not available; therefore, this field requires prodigious studies to establish a useful correlation between the research findings and clinical safety and toxicity studies. Further attempts should also be focused on improving the hydrogel design for combination therapy, controlled drug release, overcoming long-term treatment challenges, and reducing the cost of treatment.

## Figures and Tables

**Table 1:** Semi-synthetic hydrogel composites encapsulating cells and small molecule drugs for arthritis treatment.

Hybrid Hydrogel Composite	Hybrid Semi-Synthetic Hydrogel Composition		Cross-Linking Mechanism	Improved Hydrogel Properties	Hydrogel-Encapsulated Agent	Therapeutic Outcome/Disease Model	Refs.
	Natural Polymer and/or Its Modification	Synthetic Polymer and/or Its Modification					
GC/poly(EO-co-Gly)-CHO	Glycol chitosan (GC)	Poly(ethylene oxide-co-glycidol)-CHO(poly(EO-co-Gly)-CHO) (as cross-linker)	Schiff’s base formation	Hybrid hydrogel showed 3.1 times decrease in gelation time from 204 sec to 64 s and a consequently 2.4- and 7-fold increase in degradation time and strength modulus, respectively, with an increase in cross-linker concentration from 0.25 to 2.0 (wt.%).	Chondrocytes	Both in vitro and in vivo studies (ICR mice) confirmed slow degradation of hydrogel with a lifetime of more than 3 months, maintained cell morphology and chondrogenic ability.	[112]
MeHA/F127DA	Methacrylated HA (HAMA)	Pluronic F127 diacrylate (F127DA) nano-micelle	Photo-cross-linking	The optimized hybrid NMgel showed a low swelling ratio. The G′ of hybrid NMgel increased from 2 kPa to 10 kPa and a maximum of 20 kPa with increasing HAMA content from 0.25% to 0.75% and 1.5%.	Stem cells	Hybrid hydrogel efficiently supported the cartilage regeneration following 8-week post-implantation in thyroid cartilage defects in rabbits.	[113]
SF/PVCL	Silk fibroin (SF)	Poly(*N*-vinylcaprolactam) (PVCL)	Photo-cross-linking	T_sol–gel_ 32–35 °C, increased water uptake ability and higher elastic response.	Mouse pre-chondrocyte (ATDC5) cells	In vitro studies with ATDC5 cells reported enhanced chondrogenic response.	[114]
ALG-POL/SF	SF	Synthesized alginate- poloxamer 407 copolymer	Chemical cross-linking (HRP and H_2_O_2_)	The hybrid hydrogel (5.6% ALG-POL + 8% SF) exhibited a large swelling index, thermoresponsive, highly porous, and strong mechanical characteristics. Gelation time 7.5 min and G′ value ~ 5 kPa.	Chondrocyte	The hydrogel facilitated in vitro chondrocyte growth without affecting their chondrogenic phenotype.	[115]
AD/CS/SF	SF	Alginate-dopamine (AD), chondroitin sulfate-NHS (CS-NHS)	Chemical cross-linking (HRP and H_2_O_2_)	The AD/CS/SF hydrogel showed a lap shear strength of 120 kPa, with a comparable gelation time and adhesive strength (121 kPa) with that of commercial *Enbucrilate* tissue adhesive. Slow degradation with retention of 60% of mass even after 20 days in phosphate-buffer saline (PBS).	Exosomes (EXO) isolated from BMSCs	Hybrid AD/CS/SF/EXO hydrogel promoted the cartilage defect regeneration in situ, and ECM remodeling. The exosomes secreted by the hydrogels could induce the migration of BMSCs to the hydrogel and neocartilage via the chemokine signaling pathway in osteochondral defect model rats.	[116]
HA-SH/p(HPMAm-lac)-PEG	Thiolated HA (HA-SH)	Vinyl sulfonated triblock polymer: methacrylated poly[N-(2-hydroxypropyl)methacrylamide mono/dilactate]/polyethylene glycol (p(HPMAm-lac)-PEG)	Michael-type addition	Gelation temperature 20–22 °C, longer residence time with complete hydrogel degradation in 40–70 days in PBS.	-None	In vivo efficacy studies in OA mouse model exhibited reversion of inflammation-related symptoms with downregulation of TNF-α, NF-kB, and RANKL and induction of MSC maturation in to chondroblasts and cartilage formation.	[117]
HA/PLA-b-PEG (with NO-cleavable cross-linker) (DA-NOCCL)	Azide-HA (HA-N_3_)	Azide-PLA-b-PEG	Click cycloaddition (azide-alkyne reaction)	Hydrogel possessed self-healing behavior, providing visco-supplementation with dual drug (both hydrophilic and hydrophobic)-releasing features, in response to different NO concentrations.	Dexamethasone (Dex)	Intra-articular injection of dex-encapsulated, NO-scavenging hybrid hydrogel remarkably suppresses NO-mediated pro-inflammatory cytokine levels and showed superior therapeutic effects in CIA mice models.	[118]
PEG/SF hydrogel	SF	6-amino-2-cyanobenzothiazole (CBT)/bocethylmercapto-L-cysteine-functionalized 4-armed PEG (PEG-CBT/PEG-d-Cys)	Thiol based bio-orthogonal reaction and ultrasonication	Porous DN hydrogel (150 µm pore size), short gelation time of 10 sec and superior mechanical properties. PEG-SF (50:50) showed highest G′ value of 15 kPa and compressive stress 0.37 MPa compared to 2–3 kPa (G’) and 0.08 MPa with pure PEG hydrogel.	BMSCs	Hydrogel maintained in vitro BMSC viability and increased differentiation. Moreover, PEG/SF hydrogel promoted the regeneration of cartilage defects in vivo in cartilage defect SD rat model.	[119]
HA/PEG (namely DAHP)	Furan-HA (FHA)	Maleimide- PEG	Diels–Alder cross-linking	Hybrid DAHP (FHA: Mal-PEG-Mal, 1:5) showed the fastest gelation time of ~1800 s, while FHA:Mal-PEG-Mal (1:1.25 and 1:2.5) displayed gelation time of more than 1 h at 37 ℃. Hydrogel exhibited slow-release kinetics for MSC-sEVs.	Mesenchymal stem cell-derived small extracellular vesicles (MSC-sEVs)	HA/PEG Hydrogel retained the therapeutic functions of sEVs, and an in vivo test unveiled that the hydrogel could enhance the therapeutic efficacy of MSC-sEVs for OA improvement in traumatic OA rat model.	[120]
Graphene oxide (GO) doped GG/PEGDA bilayered hydrogel	Gellan gum (GG)	Poly(ethylene glycol) diacrylate (PEGDA)	Photo cross-linked and Ionic cross-linked (MgCl_2_)	Bilayered GG/PEGDA hydrogel mimicked the mechanical and lubrication features of articular superficial and deep cartilage zones with a Young’s modulus of ~300 and 700 kPa, respectively.	None-	Bilayered doped hydrogel demonstrated antiwear properties and was non-cytotoxic to human chondrocytes.	[80]
CS/PCL/KGN	Chitosan (CS)	Polycaprolactone (PCL)	Thermosensitive	Multi-layered CS/PCL/KGN hydrogel scaffold showed lesser swelling extent and greater compressive modulus, with sustained KGN release.	Kartogenin (KGN)	The scaffold promoted the proliferation and chondrogenic differentiation of laden MSCs, with increased production of type II collagen and Sox9.	[121]
PL407-PL338/HA	HA	Poloxamer 407 and 338(PL407, PL338)	Thermosensitive	Poloxamer/HA hybrid hydrogel exerted viscoelastic behavior and cubic phase organization. Gelation temperature and G′ value was determined as 32 °C and 6.6 kPa, respectively.	Sulforaphane (SFN)	The hydrogel showed non-cytotoxicity to both osteoblast and chondrosarcoma cell lines. In vitro/ex vivo experiments exhibited an increased expression of type II collagen, and proteoglycan accumulation.	[122]

**Table 2:** Nano/microcomposite integrated hydrogel composite for arthritis treatment.

Formulation (Liposome/Nanoparticles/Microspheres)	Therapeutic Agent	Hydrogel Characteristics	Characteristics of Colloidal Drug Carrier	Therapeutic Outcome	Refs.
Methotrexate entrapped ultradeformable liposomal carbopol gel	Methotrexate (MTX)	Transdermal delivery, viscosity 11847 mPa.s, gel formulation revealed a non-Newtonian pseudoplastic (shear-thinning) flow pattern	Liposome size 100 nm, high drug content 98%, skin permeation studies demonstrated permeability coefficient K_p_ values as 9.6 × 10^−3^ cm/h.	Improved anti-rheumatic activity was observed in AIA rat model, reduced expression of TNF-α and IL-1β in paw tissues, reduced edema volume improved tissue architecture and body weight gain (23%).	[150]
Carbopol hydrogel loaded with methotrexate aspasomes	Methotrexate (MTX)	Transdermal delivery, T_sol–gel_ 37 °C	Aspasome 386 nm, drug loading 19.41% and in vitro drug release for more than 24 h.	Reduced TNF-α, IL-β production, cartilage damage, inflammation, and bone resorption in AIA rat model.	[151]
Poly(N-isopropylacrylamide)(PNIPAM)/HA (HA)hydrogel containing nano/microparticles	Chondrogenic small molecule melatonin	Injectable, PNIPAm/HA hydrogel showed 50% shrinkage at equilibrium state, in vitro degradation with only 43% degradation after 40 days, compression module 109.04 kPa	Chitosan-g acrylic acid coated PLGA MPs were of size 2.1–2.2 µm, with loading content 3.4% and encapsulation efficiency 16%; NPs were 130 nm with loading content 2%, encapsulation efficiency 8%, and controlled release up to 15 days.	High chondrogenic differentiation potential (in vitro studies) for CTE.	[152]
Silk fibroin hydrogel containing chitosan NPs	Transforming growth factor-β1 (TGF-β1) and bone morphogenic protein-2 (BMP-2)	Water absorption capacity 20%, in vitro hydrogel degradation of 40% in 32 days	Chitosan NPs size 343.7 ± 20.48 nm, 75–80% TGF-β1 and 80% BMP-2 release in 15 days.	Enhanced chondrogenic ability both in vitro and in vivo (New Zealand white rabbit articular cartilage defect model).	[153]
HA hydrogel encapsulating nanocrystals	Camptothecin (CPT)	Intra-articular injection, gelation time 5 min	CPT nanocrystal size 160–560 nm, in vitro drug release up to 1 month.	Decreased cytokine level (IL-1β and IL-6) in joint homogenate. Histological and micro-CT analysis at 60 days showed joint recovery with CPT-hydrogel compared with disease control (severe joint destruction) in CIA rat model.	[154]
HA-fibrin hydrogel encapsulating nanocapsules	Dexamethasone (Dex) and galectin-3 inhibitor (GI)	Intra-articular injection, T with 29G needle, gelation time less than 30 s, hydrogel viscosity following gelation 81.3 mPa.s, and elastic behavior with G′ > G″	Dex nanocapsules size 135 ± 9 nm and GI nanocapsules were 122 ± 11 nm, 100% drug release in 24 h from nanocapsules while it took 72 h to show 100% drug release from hydrogel.	Acute synovitis CIA rat model studies reported significant increase (~40%) in knee diameter with reduction in swelling and inflammation.	[27]
Polymeric NPs hydrogel system, [carboxylic acid termini-functionalized poly(organophosphazene)CP]	Triamcinolone acetonide (TCA)	Intra-articular injection, gelation temperature 32–37 °C, exhibited high viscosity of 518.75 Pa.s at 37 °C, rheology study: G′ and G″ values of 1284 and 765 Pa, respectively, at 37 °C indicated a gel state.	Self-assembled organophosphazene NPs size 140 nm, sustained release profile up to 35 days.	Long-term anti-inflammatory effect and prevention of cartilage degeneration by inactivating MMPs were observed in MIA-induced OA rat model.	[155]
Chitosan hydrogel loaded with diclofenac-sodium-loaded alginate microspheres	Diclofenac sodium (DS)	Intra-articular administration, injectable thermosensitive hydrogel, exhibited T_sol–gel_ at 31.72 ± 0.42 °C, gelation time 5 min.	Alginate microspheres, in vitro drug release up to 5 days.	Improved anti-inflammatory efficacy in New Zealand rabbits with experimental arthritis.	[156]
Hydrogel containing methotrexate-loaded alginate microspheres	Methotrexate (MTX)	Injectable thermosensitive hydrogel, T_sol–gel_ 37 °C, gelation time 5 min, swelling degree 5.8%, G′ and G″ at 37 °C was 500 and 100 Pa, respectively, at oscillatory frequency of 1 Hz. Viscosity of sample was decreased with increased shear rate.	Non-cross-linked and cross-linked alginate MPs size 5–6 and 8 µm, respectively, high encapsulation efficiency. Fast drug release (95–98% release in 8 h) with non-cross-linked MPs while cross-linked alginate MPs showed sustained release, 75% MTX release in 66 h.	Significant decrease in swelling and paw edema following treatment with cross-linked MTX-MP-loaded-hydrogel composite with no signs of toxicity in AIA rat model.	[157]
DMA-MPC coated GelMA hydrogel microspheres	DS	Intra-articular administration, modified hydrogel microspheres	GelMA microspheres 150 µm, porous structure, loading efficiency (10–15%), DMA-MPC polymer coated microspheres demonstrated 76% degradation at 28 days.	Intra-articular injection at rat knee joint (osteoarthritic rat model) showed improved lubrication and anti-inflammatory effects with reduced expression of matrix metalloproteinase-13 and ADAMTS5.	[158]
TCA-loaded MPs in poly(polyethylene glycol methacrylate) poly(PEGMA)hydrogel	TCA	Intra-articular injection, gelation temperature 33–37 °C, viscosity 12,426 cP at shear rate of 0.5 rpm, and detachment force 6063 dyne/cm^2^	PLA/methoxy-PEG-poly(δ-decalactone) (mPEG-PDL) MPs, entrapment efficiency 84%, loading content 7.6%, and 90% TCA release in 160 h.	Percent inhibition of inflammation vs. time profile demonstrated AUC values with 450–514%/day and significantly reduced adjuvant-induced joint inflammation in rats.	[159]
MTX-loaded polyelectrolyte complexes/Poloxamer 407 and 188 hydrogels	Methotrexate (MTX)	Intra-articular injection, gelation behavior close to physiological temperature at 36.7 °C	Oligochitosan NPs (PEC) spherical in shape, size 470 nm, 50% drug release in 1 h.	Hybrid hydrogel composite exhibited reduced plasmatic IL-1β compared to free MTX group, reduced systemic exposure of MTX.	[132]

## Data Availability

Not applicable.
